# Genetic variation at the 8q24.21 renal cancer susceptibility locus affects HIF binding to a MYC enhancer

**DOI:** 10.1038/ncomms13183

**Published:** 2016-10-24

**Authors:** Steffen Grampp, James L. Platt, Victoria Lauer, Rafik Salama, Franziska Kranz, Viviana K. Neumann, Sven Wach, Christine Stöhr, Arndt Hartmann, Kai-Uwe Eckardt, Peter J. Ratcliffe, David R. Mole, Johannes Schödel

**Affiliations:** 1 Department of Nephrology and Hypertension, Universitätsklinikum Erlangen und Friedrich-Alexander-Universität (FAU) Erlangen-Nürnberg, Ulmenweg 18, 91054 Erlangen, Germany; 2 Nuffield Department of Medicine, Henry Wellcome Building for Molecular Physiology, University of Oxford, Roosevelt Drive, Oxford OX3 7BN, UK; 3 Department of Computer Science 9, Friedrich-Alexander-Universität (FAU) Erlangen-Nürnberg, Cauerstraße 11, 91058 Erlangen, Germany; 4 Department of Urology, Universitätsklinikum Erlangen und Friedrich-Alexander-Universität (FAU) Erlangen-Nürnberg, Krankenhausstraße 12, 91054 Erlangen, Germany; 5 Institute of Pathology, Universitätsklinikum Erlangen und Friedrich-Alexander-Universität (FAU) Erlangen-Nürnberg, Krankenhausstraße 8-10, 91054 Erlangen, Germany

## Abstract

Clear cell renal cell carcinoma (ccRCC) is characterized by loss of function of the von Hippel–Lindau tumour suppressor (VHL) and unrestrained activation of hypoxia-inducible transcription factors (HIFs). Genetic and epigenetic determinants have an impact on HIF pathways. A recent genome-wide association study on renal cancer susceptibility identified single-nucleotide polymorphisms (SNPs) in an intergenic region located between the oncogenes *MYC* and *PVT1*. Here using assays of chromatin conformation, allele-specific chromatin immunoprecipitation and genome editing, we show that HIF binding to this regulatory element is necessary to *trans*-activate *MYC* and *PVT1* expression specifically in cells of renal tubular origins. Moreover, we demonstrate that the risk-associated polymorphisms increase chromatin accessibility and activity as well as HIF binding to the enhancer. These findings provide further evidence that genetic variation at HIF-binding sites modulates the oncogenic transcriptional output of the VHL–HIF axis and provide a functional explanation for the disease-associated effects of SNPs in ccRCC.

## Abstract (web summary)

Genome-wide association studies have identified multiple loci associated with the risk of developing renal cancer. Here, the authors show that one of these loci generates open chromatin, which enhances the binding of HIF and HIF-mediated transactivation of *MYC*.

In clear cell renal cell carcinoma (ccRCC), but few other cancers, somatic loss-of-function mutations, chromosomal aberrations or promoter hypermethylation lead to decreased activity of von Hippel–Lindau tumour suppressor protein (pVHL). pVHL is the recognition component of an E3 ubiquitin ligase complex that targets hypoxia-inducible factor (HIF) alpha subunits to the ubiquitin–proteasome pathway. Dysfunctional pVHL therefore disrupts proteasomal degradation of HIF-α subunits (HIF-1α and HIF-2α) and increases expression of HIF target genes^1^,^2^. VHL mutations are considered to be ‘truncal' mutations in ccRCC and HIF stabilization can already be detected in early pre-cancerous lesions in tubular segments bearing biallelic *VHL* mutations within kidneys of patients with von Hippel–Lindau disease^3^. Though the reasons for the marked tissue restriction of VHL-associated cancer are unclear, genetic and epigenetic factors can influence RCC development^4^,^5^,^6^,^7^. In this context, genome-wide association studies have identified single-nucleotide polymorphisms (SNPs) that are specifically associated with renal cancer susceptibility^8^,^9^,^10^. So far, two genetic regions with ccRCC-related SNPs may have an impact on the VHL–HIF signalling axis. SNPs on chromosome 2 are located within the first intron of the *EPAS1* gene coding for HIF-2α and SNPs on chromosome 11 associate with a HIF-2-binding enhancer, which *trans*-activates the *CCND1* oncogene^11^,^12^. Recently, a novel variant rs35252396, a two base pair substitution AC>CG, has been detected on chromosome 8q24.21 (ref. 9). rs35252396 is strongly associated with renal cancer risk in Icelandic and other populations of European descent (odds ratio 1.27, *P*-value 5.4 × 10^−11^, minor allele frequency 0.46 in the combined analysis)^9^. The index polymorphism is located in an intergenic and putative regulatory region interposed between the major oncogenic driver *MYC* (136 kb upstream) and the oncogenic long non-coding RNA *PVT1* (14 kb downstream). MYC orchestrates metabolic and growth-promoting pathways, and dysregulation is a hallmark of tumour initiation^13^,^14^. With respect to the VHL–HIF axis in ccRCC, MYC interacts differentially with the HIF-1α and HIF-2α subunits, thereby possibly contributing to the isoform-specific effects that are important in ccRCC^15^,^16^. Across all cancers, the *MYC* locus displays the highest susceptibility to somatic copy-number gains and both, *MYC* and *PVT1*, are co-amplified in most cases (>98%)^17^,^18^. In cancer tissue, PVT1 RNA levels correlate well with MYC levels and appear to be necessary for MYC protein stabilization and tumour growth^18^,^19^. In addition to chromosomal rearrangements, the 8q24 locus is a hot spot for intergenic SNPs associated with a variety of tumours such as colorectal, ovarian, urinary bladder or prostate cancer, Hodgkin's lymphoma and chronic lymphocytic leukaemia^20^,^21^,^22^,^23^,^24^,^25^,^26^,^27^,^28^,^29^,^30^,^31^,^32^,^33^. Despite this high density of multiple cancer-associated variants in the 8q24.21 region, functional analyses have mainly been restricted to the SNP rs6983267, which is associated with colorectal and prostate cancer. This SNP resides in a regulatory element 335 kb telomeric of *MYC* and influences the expression of *MYC* by affecting activity of an enhancer^34^,^35^,^36^,^37^,^38^. However, the renal cancer-associated variant rs35252396 observed in the Icelandic population is not in linkage with any other disease-associated SNP in the 8q24.21 region (*r*^2^<0.02) (ref. 9), suggesting the involvement of renal cancer-specific mechanisms in generating the predisposition.

## Results

### MYC and PVT1 are direct targets of HIF in ccRCC

**Figure 1:**
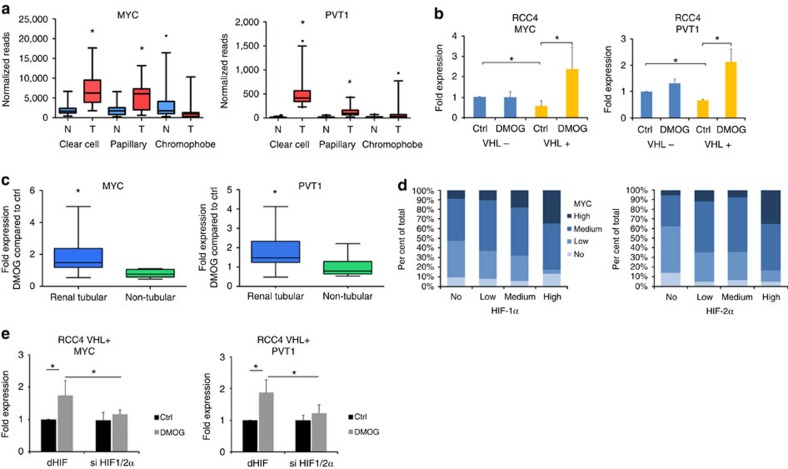
MYC and PVT1 regulation in renal cancer. (**a**) Analyses of TCGA renal cancer data sets indicate significant overexpression of MYC and PVT1 RNA in clear cell and papillary RCC. Box-and-whisker plots of normalized RNA-seq reads from matching normal (N) and tumour tissue (T) in clear cell (*n*=22), papillary (*n*=31) and chromophobe (*n*=25) RCC. Horizontal lines represent medians, whiskers the 5 and 95% percentiles and dots outliers. *Paired *t*-test, *P*<0.0001. (**b**) MYC and PVT1 RNA expression is reduced by pVHL re-expression in RCC4 renal cancer cells. HIF stabilization by DMOG induces expression of both genes specifically in RCC4 pVHL re-expressing cells. RCC4 cells with defective (blue) or functional (yellow) pVHL were exposed to vehicle or 1 mM of the HIF stabilizer DMOG for 16 h. *n*=3 from independent experiments; values are mean±s.d., *One-sample *t*-test, *P*<0.05. (**c**) Box-and-whisker plot of relative expression levels (fold change compared to untreated control) of MYC and PVT1 RNA in a collection of 10 pVHL-competent renal tubular derived cells (RCC, primary renal tubular and immortalized renal tubular cells, blue) and 10 non-tubular-derived cells (renal podocytes and non-renal cells, green) exposed to 1 mM DMOG for 16 h. For details of cell lines, please see Supplementary Fig. 2; horizontal lines represent median values and whiskers show the 5 and 95% percentiles; *Mann–Whitney *U*-test; *P*<0.05. (**d**) MYC protein levels are high in HIF-positive tumours. A total of 330 ccRCC tumours were stratified according to the immunoreactive score for HIF-1α, HIF-2α and MYC staining in no, low, medium or strong staining. Fractions of MYC signals are shown for each subgroup for HIF-staining. *χ*^2^-test; *P*<0.001 for both HIF-1α and HIF-2α. (**e**) MYC and PVT1 RNA expression in HIF-α depleted (si HIF 1α/2α, siRNA against HIF1α/2α) or control transfected (si dHIF, siRNA against drosophila HIF) RCC4 pVHL-competent cells exposed to vehicle (ctrl, black) or 1 mM DMOG (grey) for 16 h. Data present mean±s.d. from six independent experiments. *One-sample *t*-test, *P*<0.05).

In contrast with many other cancers, copy-number gains at the MYC locus are relatively infrequent in ccRCC^18^. As a first step in analysing mechanisms associated with variant rs35252396, we therefore sought to define the frequency of dysregulated MYC and PVT1 expression in renal cancer. Analysis of RNA-seq data from clear cell, papillary and chromophobe RCCs generated by the TCGA consortium^39^,^40^,^41^ confirmed that MYC and PVT1 RNA are commonly overexpressed in ccRCC (Fig. 1a). In line with the results from RNA analyses, positive MYC protein staining was strongly associated with the clear cell phenotype in a tissue microarray containing 453 unselected renal cancer specimens (Erlangen RCC Cohort, Supplementary Fig. 1). To better understand this contrast between RNA and copy-number changes, we proceeded to investigate direct involvement of the VHL–HIF axis in MYC and PVT1 expression. In pVHL re-expressing RCC4 cells with low HIF, levels of both transcripts were reduced compared with the pVHL-defective parental cells (Fig. 1b). When exposed to the hypoxia mimetic and HIF stabilizer dimethyl oxalylglycine (DMOG), we measured an increase of MYC and PVT1 RNA in pVHL re-expressing RCC4 transfectants, whereas no difference was determined in the respective pVHL-defective cells (Fig. 1b), suggesting that MYC is regulated by the HIF pathway in this context. To examine the specificity of hypoxic MYC and PVT1 regulation, we expanded our analysis to a variety of cells from renal tubular origins (renal cancer cell lines, primary renal tubular cells and immortalized renal tubular cells) and non-tubular origins (immortalized podocytes and non-renal cells) with functional re-expressed or wild-type VHL. Strikingly, significant induction of both MYC and PVT1 RNA by DMOG was specifically observed in renal cancer cell lines and tubular cells (Fig. 1c; Supplementary Fig. 2). The striking selectivity of MYC messenger RNA regulation by the VHL–hypoxia pathway in renal tubular cells was also concordant with the data from transgenic animals with conditional knockout of *VHL* in tubular cells in which MYC is strongly induced (Supplementary Fig. 3). Taken together, this suggests that genes encoding *MYC* and *PVT1* are targets of HIF in renal tubule-derived cells.

**Figure 2:**
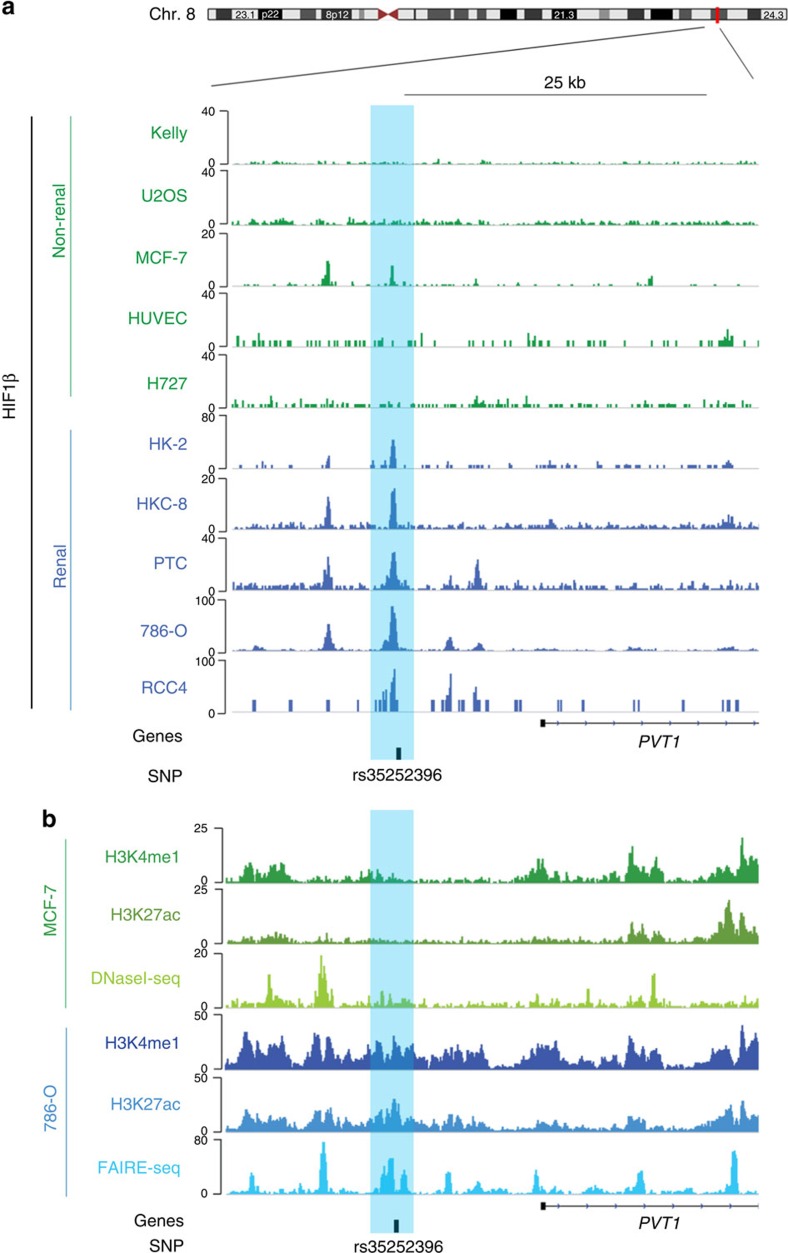
HIF-dependent MYC and PVT1 induction in ccRCC. (**a**) HIF-1β ChIP-seq signals at the 8q24.21 renal cancer susceptibility locus in non-renal cells (green) or renal tubule-derived cells (HK-2, HKC-8: immortalized tubular cells, PTC: primary tubular cells, ccRCC: 786-O and RCC4, blue). In non-ccRCC cells, HIF was stabilized by hypoxia or DMOG for 16 h. rs35252396 is located close to the HIF peak (highlighted in light blue) ∼14 kb upstream of the transcriptional start site of PVT1. (**b**) ChIP-seq (H3K4me1 and H3K27ac), DNaseI-seq and FAIRE-seq signals in MCF-7 breast cancer and 786-O renal cell carcinoma cells reveal features of active and open chromatin at the 8q24.21 SNP-associated HIF-binding site (highlighted in light blue) in 786-O cells, but not MCF-7 cells.

To test for association between MYC and HIF protein expression, we stained tissue microarray sections from the Erlangen RCC cohort for HIF-1α and HIF-2α. In ccRCC, HIF-1α and HIF-2α correlated significantly with positive MYC staining (Fig. 1d). To directly examine the role of HIF in MYC/PVT1 regulation, we performed short interfering RNA (siRNA)-mediated knockdown of HIF-α subunits in pVHL-competent RCC cells. In pVHL re-expressing RCC4 or 786-O and VHL wild-type RCC L34 cells, induction of MYC and PVT1 by DMOG was significantly reduced after HIF depletion (Fig. 1e; Supplementary Fig. 4). HIFs are transcription factors that activate gene expression by direct binding to chromatinized DNA^42^,^43^. Therefore, we interrogated both newly acquired and previously published HIF-1β chromatin immunoprecipitation–DNA sequence (ChIP-seq) data sets at the *MYC* and *PVT1* loci for HIF–DNA binding in a variety of cell types^11^,^43^. This revealed robust HIF-binding signals across a series of pVHL-defective renal cancer cell lines as well as immortalized proximal tubular and primary tubular cells in which HIF was stabilized by hypoxia or DMOG at intergenic sites located between the *MYC* and *PVT1* genes (Fig. 2a). In line with the lack of hypoxic gene induction, no significant HIF-binding signals were detected at these sites in cells not derived from renal tubules. Very interestingly, consistent HIF-binding signals in the renal tubule-derived cells almost precisely coincided with the renal cancer susceptibility SNP rs35252396, which locates 205 bp downstream of a hypoxia-responsive element (HRE) centred on the HIF-binding peak.

To address the importance of this locus in renal oncogenesis, we analysed the function of the SNP-associated HIF-binding site in detail. Inspection of epigenetic data from our laboratories and the ENCODE consortium revealed enriched signals for open (FAIRE, formaldehyde-assisted isolation of regulatory elements) and active chromatin (H3K4me1 and H3K27ac) at this site in pVHL-defective 786-O cells that are homozygous for the risk allele at rs35252396 (Fig. 2b). Confirming the cell-type specificity, levels of these markers of active chromatin were low in MCF-7 breast cancer cells that lack both HIF-α binding at the SNP-associated site and regulation of MYC or PVT1 RNA by hypoxia (Fig. 2b). As with studies of gene expression, we expanded the analysis of chromatin accessibility at the enhancer to panels of cells from tubular and non-tubular origins. In FAIRE experiments, enrichment of open chromatin at the HIF-binding locus was significantly greater in renal tubule-derived cells compared with non-tubular cells (Supplementary Fig. 5). Taken together, the data from both expression and ChIP-seq studies suggest that HIF binds at an enhancer located between the *MYC* and *PVT1* locus, and likely drives transcription of these genes. Open chromatin, HIF-binding and HIF-dependent regulation of MYC and PVT1 appear to be highly cell-type specific and restricted to cells of renal tubular origins.

### The SNP-associated enhancer is necessary for MYC regulation

**Figure 3:**
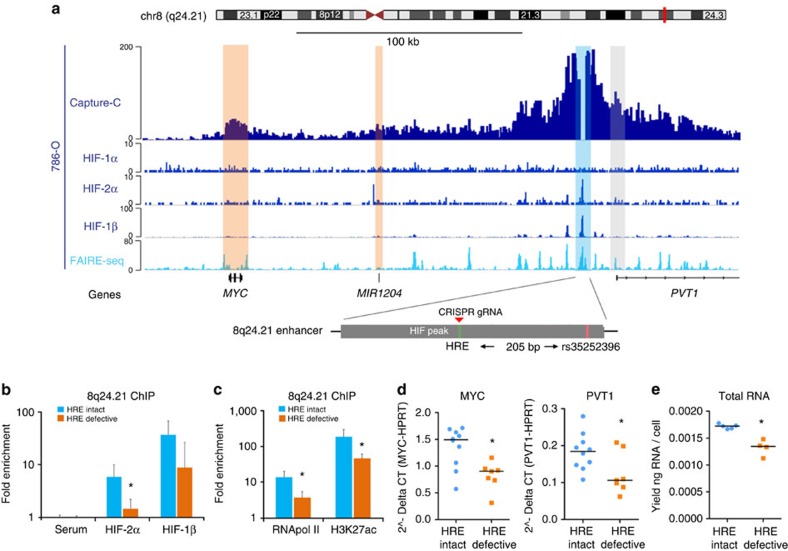
Functional analysis of the RCC associated 8q24.21 enhancer. (**a**) Capture-C assay reveals chromatin interactions between the 8q24.21 enhancer (anchor site, highlighted in light blue) and the MYC promoter (significant interactions highlighted in light orange) in 786-O cells. Interactions were also observed with the PVT1 promoter, but did not reach statistical significance (highlighted in grey). HIF ChIP-seq shows HIF-1β and HIF-2α binding in 786-O cells that lack functional HIF-1α. Positions of the central HRE, the associated SNP rs35252396 (205 bp downstream of the HRE) and the guide RNA used in CRISPR/Cas9 experiments are shown below the tracks. (**b**) HIF ChIP–qPCR confirmed reduced binding of HIF-2α (*Student's *t*-test, *P*<0.05) and HIF-1β (Student's *t*-test, *P*=0.09) to the 8q24.21 enhancer in 786-O HRE-defective cells. Data are shown in a log scale and are mean±s.d. from three independent clones of cells with intact or defective HRE, respectively. (**c**) qPCR using RNApol2 and H3K27ac ChIP samples reveals lower levels of marks of active chromatin at the 8q24.21 enhancer in 786-0 HRE-defective cells. Data are shown in a log scale and are mean±s.d. from three independent clones of cells with intact or defective HRE. *One-sample t-test, *P*<0.05. (**d**) RNA levels of MYC and PVT1 in 786-O cells with defective HRE (*n*=7) or intact HRE (*n*=10). Values represent mean from three independent RNA isolations for each clone. *Student's t-test, *P*<0.05, comparing the two groups of cells. (**e**) Total RNA levels are lower in HRE-defective cells. Each dot represents the mean yield of RNA per cell calculated from two to three independent RNA isolations from 50.000 cells per clone of cells. Values are from four (HRE-defective) or five (HRE-intact) clones of cells. *Student's *t*-test, *P*<0.05.

To further validate a functional relation between this HIF-binding enhancer and the promoters of nearby genes, we conducted chromatin conformation (Capture-C) and genome editing experiments in pVHL-defective cells. The Capture-C assay examines physical interaction of selected anchor sites with any distant chromatin region^44^. Using this technique, we observed chromatin interactions of the HIF-binding region with both the *MYC* and *PVT1* promoters in 786-O cells (Fig. 3a; Supplementary Fig. 6). However, since we had identified more than one HIF-binding signal in the ∼20 kb stretch of DNA surrounding the putative enhancer (Fig. 2a), we wished to specifically analyse the transactivation potential of the HIF binding that was most clearly associated with the predisposing SNPs. We therefore designed a guide RNA targeting the HRE in the centre of the SNP-associated HIF-binding signal and disrupted this HRE in 786-O renal cancer cells using CRISPR/Cas9 technology^45^. We screened 36 clones of cells for indel mutations at this site by PCR amplification followed by polyacrylamide gel analysis and identified 7 clones of cells with mutations that affected the HIF-binding site (Supplementary Fig. 7). We confirmed reduced binding of HIF and decreased marks of activity at the 8q24.21 enhancer in a selection of these cells with a defective HRE using HIF, RNApol2 and H3K27ac ChIP–quantitative PCR (qPCR; Fig. 3b,c; Supplementary Fig. 8). Further confirming the transactivation potential of this site, MYC and PVT1 RNA expression was significantly reduced by 40% and 32%, respectively, in comparison with the control HPRT gene, in cells with mutations that affected the HRE when compared with non-mutant clones of cells (Fig. 3d). MYC overexpression acts as a global amplifier of gene expression in tumour cells^46^,^47^. To test for the effects of reduced MYC levels on total RNA content in our cells, we measured RNA levels of HRE-defective and control cells. In line with the hypothesis of MYC-induced global gene expression, HRE-defective cells with lower MYC levels had lower RNA content than control cells (Fig. 3e). Thus, results from epigenetic analyses, Capture-C and genome editing indicate that this enhancer site interacts with *MYC* and *PVT1* promoters, is necessary for HIF-mediated transactivation of both genes and influences downstream effects of MYC.

### rs35252396 affects chromatin accessibility and HIF binding

**Figure 4:**
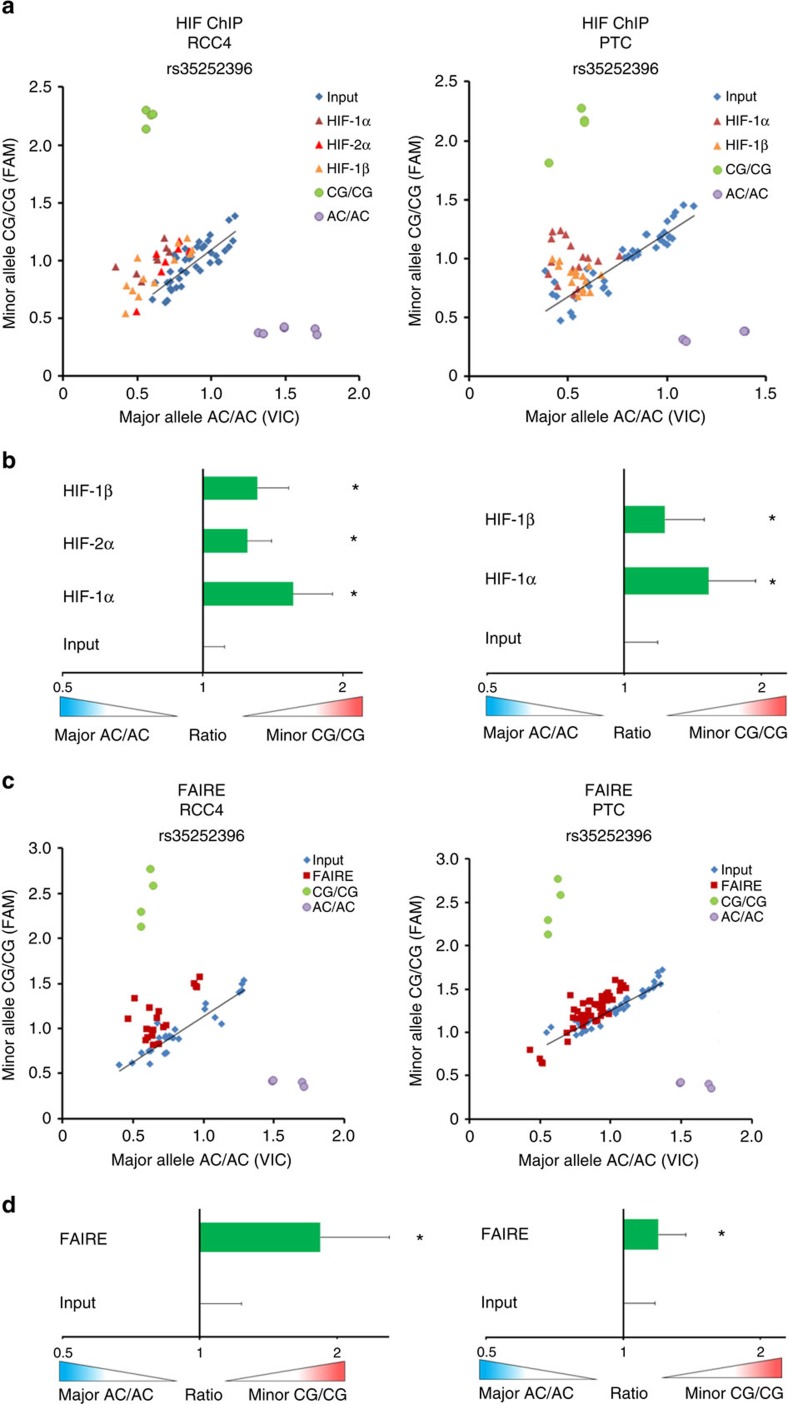
Polymorphisms at 8q24.21 affect HIF binding and chromatin accessibility. ChIP and FAIRE assays in cells that are heterozygous for SNP rs35252396. (**a**) Allele-specific qPCR for rs35252396 using DNA samples from RCC4 (left) and primary renal tubular (PTC, right) cells derived from HIF-1α, HIF-2α (only in RCC4) and HIF-1β ChIP experiments. Input DNA was used as a control and genomic DNA from homozygous cell lines was used for positive controls for the alleles (AC/AC-Caki1 cells and CG/CG-786-O cells). A shift of the qPCR signals towards the minor allele CG indicates enrichment for the risk allele. Data are from three independent ChIP experiments (RCC4) or one ChIP experiment per PTC culture exposed to DMOG for 16 h from four different individuals. (**b**) Ratios of qPCRs signals from **a**. The mean ratio of minor allele CG/major allele AC for the input DNA (before immunoprecipitation) was arbitrarily set to 1. Data are mean±s.d.. Data are from three independent ChIP experiments (RCC4) or one ChIP experiment per PTC culture exposed to DMOG for 16 h from four different individuals (three to four technical replicates for each experiment). *One-sample *t*-test, *P*<0.05. (**c**) Allele-specific qPCR for rs35252396 using FAIRE and input DNA samples from RCC4 (left) and primary renal tubular (PTC, right) cells. Results are from two independent experiments (RCC4) or one FAIRE experiment per PTC culture from four different individuals. DNA from homozygous cell lines was used for positive controls for the homozygous alleles (AC/AC and CG/CG). Signals of the positive controls for the homozygous alleles are similar in RCC4 (left) and PTC (right) because these samples were analysed by allele specific in the same qPCR experiment. (**d**) Ratios of qPCRs signals from **c**. The mean ratio minor allele CG/major allele AC for the input DNA was arbitrarily set to 1. Data are mean ±s.d. from two independent experiments (RCC4) or one FAIRE experiment per PTC culture from four different individuals (8–10 technical replicates each). *One-sample *t*-test, *P*<0.05.

The overlap of a HIF-binding enhancer and renal cancer predisposing SNPs led us to consider whether the SNPs could affect HIF–DNA interactions and enhancer activity. In reporter assays using a DNA sequence spanning the HIF-binding site and SNPs, we measured a significant hypoxic induction of the reporter gene but no effect of the SNPs on reporter gene activity or hypoxic induction (Supplementary Fig. 9). Reporter assays use non-chromatinized DNA and therefore do not capture epigenetic effects caused by the action of SNPs in chromatinized DNA. To test for such an action, we proceeded to assays that examine SNP-associated effects on native chromatin. rs35252396 is located 205 bp downstream of the HIF-binding HRE and thus is likely to be present in most of the DNA fragments that are immunoprecipitated with HIF antibodies from native chromatin or isolated by FAIRE. We reasoned that this would allow testing of captured DNA for allelic imbalance of rs35252396 with respect to HIF binding or open chromatin. For these experiments, we identified primary tubular cells as well as pVHL-defective RCC4 and RCC L13 renal cancer cells that are heterozygous for rs35252396. In genotype-specific qPCR assays, the risk allele at rs35252396 was significantly enriched in chromatin fragments that bound HIF and had marks of active chromatin (Fig. 4a,b; Supplementary Figs 10 and 11). To validate this data, we sequenced amplicons from ChIP experiments in RCC4 cells, which resulted in a similar allelic imbalance as obtained with qPCR assays (Supplementary Fig. 12). HIF binding to HREs is highly dependent on the presence of open chromatin at regulatory DNA elements^43^. We thus performed FAIRE experiments in the heterozygous cells to test for allelic imbalance in open chromatin and measured a consistent imbalance towards the risk allele at rs35252396 in the DNA fragments recovered by FAIRE compared with input DNA. Similar results were obtained in both RCC cell lines and primary renal tubular cells (Fig. 4c,d; Supplementary Fig. 13). These data indicate that differential HIF binding at the renal cancer-associated 8q24.21 MYC/PVT1 enhancer is affected by SNPs that modify accessibility and activity of this site.

### Allelic imbalance in MYC and PVT1 expression

**Figure 5:**
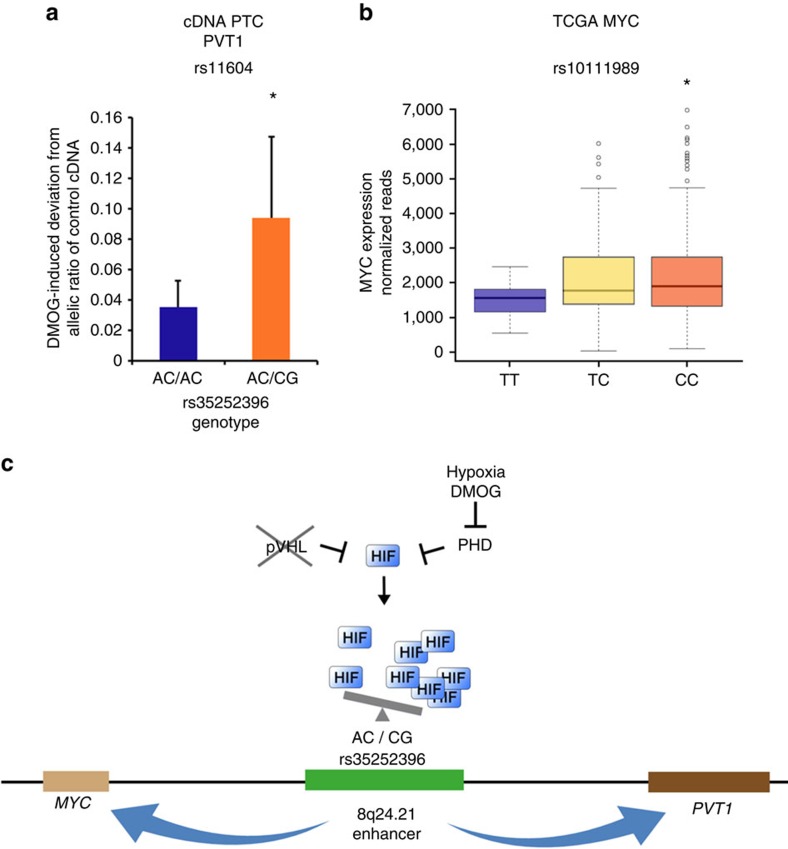
Polymorphisms at 8q24.21 influence expression of MYC and PVT1. (**a**) HIF stabilization leads to allelic imbalance in PVT1 expression. Twelve primary renal tubular cell cultures (PTC) were identified that are heterozygous for SNP rs11604 that resides in the coding region of *PVT1*. Four individuals had an AC/AC genotype and eight individuals had an AC/CG genotype at rs35252396. For rs11604, the ratio of allele-specific signals was measured by qPCR (FAM/VIC) from input genomic DNA and cDNA derived from control (untreated) or DMOG-treated cells. No significant allelic imbalance at rs11604 was detected between the two groups (AC/AC or AC/CG at rs35252396) in input genomic DNA or cDNA from untreated cells (Supplementary Fig. 14). We then calculated the change of allelic ratios of cDNA from DMOG-treated cells compared with the respective control untreated cDNA. We detected a significant greater change in allelic PVT1 expression induced by DMOG in cells from individuals with an AC/CG genotype at rs35252396. Values are mean±s.d. from four (AC/AC) or eight (AC/CG) individuals. qPCRs for DNA and cDNA were performed in triplicates per individual. *Student's *t*-test, *P*<0.05. (**b**) Genotype expression correlation for rs10111989 and MYC in the KIRC TCGA data. rs10111989 is in LD with rs35252396 (*r*^2^=0.33, D′=0.98) and allele C is associated with RCC development (odds ratio 1.16, *P*<0.05) in data from a meta-analysis of UK and NCI cohorts^54^. *χ^2^-test; *P*<0.05 for higher expression in CC individuals compared with TT individuals. Whiskers extend to 1.5 times of the inter quartile range. (**c**) Schematic representation of the 8q24.21 RCC enhancer of MYC and PVT1 expression. In cells from renal tubular origin (non-transformed tubular cells or RCC cells), HIFs can bind to the enhancer and regulate MYC and PVT1 expression, but binding is dependent on the rs35252396 genotype that affects accessibility of the site.

Allele-specific HIF binding should result in an allelic imbalance in MYC and PVT1 expression. To examine this, we identified primary renal tubular cells from 12 individuals who are heterozygous for rs11604, a SNP in the coding region of PVT1. This SNP is in weak LD with rs35252396 (*r*^2^=0.049; D′=0.270) and therefore the phase in the primary renal tubular cells is not known. We measured the allelic balance of rs11604 in genomic DNA and cDNA from control (untreated) or DMOG-treated cells using genotype-specific qPCR assays. Allelic imbalance in control samples was comparable between cells from individuals with a heterozygous (*n*=8) and homozygous (*n*=4) genotype at rs35252396 (Supplementary Fig. 14). However, the change in allelic expression of PVT1 induced by HIF stabilization compared with control was significantly greater in cells from individuals with a heterozygous genotype at rs35252396 (Fig. 5a). This is consistent with the hypothesis that unequal HIF binding at the enhancer drives differential expression of PVT1 from the two alleles. We were unable to establish a similar assay for the MYC-coding region and therefore resorted to genotype and tumour gene expression data from the TCGA consortium. rs35252396 was not genotyped in this cohort, but we identified SNPs in the TCGA cohort (rs10111989, rs4733579 and rs17775239) that were genotyped and are in LD with rs35252396 (ref. 9). Analysis of genotype expression correlations revealed that the risk allele of SNP rs10111989 (pairwise LD with rs35252396: *r*^2^=0.33, D′=0.98 (ref. 9)) exhibited a significant association with higher MYC expression (*χ*^2^-test, *P*=0.0296, Fig. 5b). The other SNPs showed a correlation with MYC expression, but did not reach statistical significance (Supplementary Fig. 15). Though this analysis itself cannot implicate any specific polymorphism in the regulation of MYC in renal cancer, it is consistent with the data above implicating an rs35252396-associated phenotype.

Taken together, our findings identify a HIF-binding enhancer of oncogenic MYC and PVT1 expression (Fig. 5c). HIF binding, activity and accessibility of the enhancer as well as MYC/PVT1 induction are restricted to cells from renal tubular origin and dependent on the genotype of rs35252396, a polymorphism associated with renal cancer susceptibility.

## Discussion

We demonstrate here that unrestrained activation of HIF in pVHL-defective renal cancer enhances expression of MYC and PVT1 via long distance interactions with a HIF-binding intergenic enhancer. This regulatory potential already exists in non-transformed tubular cells and in our analysis appears to be restricted to renal tubular cells. The pathway might therefore be important for cell homeostasis even in normal tubular epithelium and the earliest tubular neoplastic lesions that arise following inactivation of VHL. Modulation of HIF activity and MYC/PVT1 expression by polymorphisms located in this intergenic region could then promote or retard renal tumorigenesis. That many of the currently known renal cancer-associated SNPs (*EPAS1*, *CCND1* and *MYC*/*PVT1*) can be linked to modulation of a single pathway (that is, the HIF pathway) is striking and to our knowledge unique in tumour biology. Interestingly, analysis of two of these loci, *CCND1* and *MYC/PVT1*, indicates that the susceptibility determinants appear to operate on the chromatin structure at tissue-specific HIF-binding loci^11^. We conclude that the predisposing or protective effects of renal cancer-associated polymorphisms are explained at least in part by their ability to promote or inhibit, respectively, HIF expression or HIF-mediated transactivation of key oncogenic pathways.

## Methods

### Cell culture

HKC-8 cells were provided by L. Racusen and 786-O cells re-expressing pVHL were a gift from W.G. Kaelin Jr. RCC10, RCC L13 and RCC L34 cells were from M. Wiesener. RCC4 cells were a gift from C.H. Buys. HK-2, HUH7, SkHep1 and Kelly cells were from C. Warnecke. The human podocyte cell line was from M. Saleem and P. Mundel. Human umbilical vein endothelial cells (HUVEC) cells were a gift from colleagues of the Department of Cardiology, University of Erlangen-Nürnberg. 786-O, I Hela, MCF-7, Hep3B, HT1080, HepG2, HEK293, H727 and U2OS) were purchased from American Type Culture Collection. Cell lines were grown in DMEM, 100 U ml^−1^ penicillin, 100 μg ml^−1^ streptomycin and 10% fetal bovine serum (Sigma Aldrich). HKC-8 cells were cultured in DMEM/Ham's F-12 supplemented with 10% fetal calf serum, 2 mM L-glutamine, 100 U ml^−1^ penicillin and 100 μg ml^−1^ streptomycin, 5 μg ml^−1^ insulin, 5 μg ml^−1^ transferrin, and 5 ng ml^−1^ selenium (Sigma Aldrich). Healthy human kidney cortical tissue from patients undergoing tumour nephrectomy was used for tubular cell isolation. Informed consent was given by each patient and the use of the tissue was approved by the local ethical committee at the University of Erlangen-Nürnberg. After mincing and DNAse I (Roche Diagnostics) and collagenase II (Gibco) digest, cells were sieved through a 100 μm and a 70 μm filter. Primary human tubular cells were cultured in DMEM/Ham's F-12 supplemented with 10% fetal calf serum, 2 mM L-glutamine, 100 U ml^−1^ penicillin and 100 μg ml^−1^ streptomycin, 5 μg ml^−1^ insulin, 5 μg ml^−1^ transferrin, 5 ng ml^−1^ selenium (Sigma), tri-jodothyronin (T3) 10 ng ml^−1^, hydrocortisone 1 mg ml^−1^, and epidermal growth factor 100 μg ml^−1^ (Peprotech). Epithelial origin was confirmed by immunocytochemistry for N- and E-Cadherin. HT1080 cells were cultured in minimal essential medium 10% fetal calf serum, 2 mM L-glutamine, 100 U ml^−1^ penicillin and 100 μg ml^−1^ streptomycin. As indicated sub-confluent cell cultures were exposed to 1 mM DMOG (Cayman) before collecting.

### Chromatin immunoprecipitation

ChIP experiments were performed using the Upstate protocol (Millipore). Two 15 cm dishes of sub-confluent cells were used for crosslinking (1% formaldehyde for 12 min on ice). After 5 min incubation with glycine 125 mM on ice, cells were lysed in 1 ml lysis buffer and sonificated using a Bioruptor Plus sonicator (Diagenode) using 28 cycles with 15 s on and 15 s off. For immunoprecipitations, 6–10 μl of antibodies against HIF-1α (rabbit polyclonal, PM14 or Cayman Chemicals, Cay10006421), HIF-2α (rabbit polyclonal, PM9 or PM8), HIF-1β (rabbit polyclonal, Novus Biologicals, NB100-110), RNA polymerase II (rabbit polyclonal, Santa Cruz, SC-899), H3K27ac (rabbit polyclonal, Abcam, ab4729), H3K27ac (rabbit polyclonal, Diagenode, pAb-174-050), H3K4me1 (rabbit polyclonal, Abcam, ab8895) and H3 (rabbit polyclonal, Abcam, ab1791)^11^,^43^,^48^,^49^ were used per 150 μl cell lysate. Rabbit serum or IgG (Millipore, 12–370) were used as negative controls as appropriate. Antibody–chromatin complexes were pulled down by proteinase A agarose beads (Millipore). After reversal of the crosslinking by heat, DNA was isolated by phenol–chloroform extraction. For ChIP–qPCR experiments, primers spanning the HIF-binding site within the 8q24.21 enhancer, a positive control at an *EGLN3* intronic enhancer and a negative control at the *CCND1* locus were used. qPCRs were performed using SYBRgreen chemistry (Thermo Scientifc) on a Step-one plus real-time PCR cycler (Applied Biosystems). Primers are listed in Supplementary Table 1.

### siRNA transfection and RNA isolation

siRNA against HIF-1α, HIF-2α and dHIF (drosophila HIF, control) are listed in Supplementary Table 2 and have been described earlier^50^. siRNA was transfected using Saint red (Synvolux) transfection reagent at a final concentration of 40 nM. Transfection was repeated after 24 h and cells were collected 48 h after the first transfection. Total RNA from cells or tissue was isolated using Tri Reagent (Sigma Aldrich) or peqGold total RNA kit (PeqLab) according to the manufacturer's protocol and transcribed into cDNA using the high capacity cDNA reverse transcription kit (Life Technologies). qPCRs were performed as described above and primers are listed in Supplementary Table 3.

### Immunoblotting

Cells were lysed in urea/SDS buffer and proteins were resolved by SDS–polyacrylamide gel electrophoresis. After transferring the proteins onto polyvinylidene difluoride membranes, HIF or MYC proteins were detected using anti-HIF-1α (1:1,000, rabbit polyclonal, Cayman Chemicals, Cay10006421), anti-HIF-2α (1:1,000 goat polyclonal, R/D, AF2997) and anti-MYC (1:1,000, rabbit monoclonal (Y69), Abcam, ab32072) antibodies. In addition, anti-β-actin (1:10,000, mouse monoclonal (AC-74), Sigma Aldrich, A5316) and horseradish peroxidase-conjugated anti-rabbit (1:10,000), anti-goat (1:2,000) or anti-mouse (1:10,000) secondary antibodies (Dako) were used. Representative western blots are shown in Supplementary Fig. 16. Immunoreactive bands were quantified using the ImageQuant TL 8.1 software (GE Healthcare) and normalized to signals for β-actin.

### Formaldehyde-assisted isolation of regulatory elements (FAIRE)

Following the protocols from Giresi *et al*.^51^ with some modifications, two 15 cm dishes of sub-confluent cells were used for crosslinking (1% formaldehyde for 5 min at room temperature) and for the preparation of input DNA. The isolation of DNA was performed using three rounds of phenol–chloroform extraction. SYBRgreen qPCR was performed on FAIRE DNA and input DNA. Values were normalized to input DNA and compared with a region just outside of the putative regulatory region. Primer sequences are listed in Supplementary Table 4.

### Capture-C assay

Experiments were performed according to Davies *et al*.^44^. 3C libraries generated from 786-O cells with DpnII and were sonicated to 200 bp. Indexed libraries were generated with NEBnext reagents (#E6000, #E7335, New England Biolabs). Capture enrichment was performed with the SeqCap EZ system (#06953212001, Roche/Nimblegen) following the manufacturer's instructions. An amount of 1–2 μg of indexed library was incubated with 13 pmol of a pool of biotinylated oligos (Intergrated DNA technologies or Sigma). A double capture protocol was followed with 48 and 24 h hybridizations^44^,^52^. Capture efficiency was determined with qPCR relative to a standard curve of genomic DNA before sequencing.

### DNA extraction

DNA was isolated using DNA cell lysis buffer (NaCl 100 mM, Tris pH 8.0 10 mM, EDTA 25 mM, SDS 0.5%, Proteinase K 0.1 mg ml^−1^) for 1 h at 45 °C. After proteinase K digest, the isolation of DNA was performed with phenol–chloroform and salt precipitation. DNA content was measured by NanoDrop (Peqlab).

### High-throughput sequencing

ChIP-seq library preparations were carried out using Illumina protocols and libraries were sequenced on a HiSeq 2000 platform (Illumina). Sequences were mapped to NCBI build 37 (hg19) using BWA and peaks were called with MACS as previously described^53^. Capture-C libraries were sequenced on the HiSeq 4000 (Illumina). Capture-C data were analysed as previously described^52^. In brief, reads were trimmed, *in silico* digested for DpnII and aligned to the GRCh37 (hg19) with Bowtie 1.0 and interaction frequencies were determined using CCanalyser2.pl (ref. 44). Significant interactions were called using a background model of distance-dependent decay from the capture site, interaction frequencies above the background level were analysed for significance^52^. For ChIP–PCR amplicon sequencing barcoded PCR, primers spanning a 329 bp sequence at the 8q24.21 enhancer (hg19: chr8:128889122-128889450) were designed. PCR products from RCC4 ChIP samples were amplified and libraries prepared using Illumina protocols. Sequencing was performed on a Miseq sequencing system (Illumina). Sequences from the different samples were decoded and analysed for the presence of SNP rs35252396.

### Expression quantitative trait loci (eQTL) association and significance

We used data from a meta-analysis of UK and NCI RCC cohorts to identify risk variants of SNPs in the 8q24.21 region that are in LD with rs35252396 (C for rs10111989, *P*<0.05; G for rs4733579, *P*<0.01; A for rs17775239)^9^,^54^. TCGA level 3 RNA-Seq expression for 450 ccRCC patients was coupled with their Affymetrix Genome-Wide Human SNP Array 6.0 level 2 data. SNP reference rs10111989 was mapped to SNP_A-8366368, rs4733579 to SNP_A-8558309 and rs17775239 to SNP_A-8682521 in the Affymetrix array. For SNP rs10111989, we excluded patients with low-confidence (>0.01) calling of the SNP status, including only 363 patients. To identify significance of association between the SNP genotype and the expression of both MYC and PVT1, RNA-seq expression across the patients was fitted to a negative binomial Generalised Linear Model against the genotype status. We then computed the likelihood ratio of this model versus a model that ignores genotype status. Finally, we used *χ*^2^-test to call significance of the genotype coefficients in stratifying the patients.

### Luciferase reporter assays

A 329 bp (hg19: chr8:128889122-128889450) insert was PCR amplified from genomic DNA and cloned into the pGL3 promoter construct (Promega) using KPNI and NHEI restriction sites. Primers are listed in Supplementary Table 5. Transfections of plasmids (1 μg per well) were performed in Hela cells using X-treme gene transfection reagents (Roche). Cells were cultured in a 24-well plate, transfected with the plasmids at 30–50% cell density and stimulated with DMOG (1 mM) or vehicle for 16 h. Cells were collected 24 h after transfection. Luciferase activity was measured using a Luciferase reporter system (Promega). Cells were co-transfected with a plasmid expressing β-galactosidase and luciferase activity was normalized to the activity of β-galactosidase. All constructs were sequence verified.

### Genome editing

For genome editing, the Gene-Art CRISPR Nuclease Vector kit (Life Technologies) was used. The guide RNA was designed according to the algorithms provided by the Zhang lab (http://crispr.mit.edu/)^45^. The guide has a quality score of 93. The CRISPR-Cas9 plasmid was cloned following the manufacturer's protocol. A total of 2 × 10^7^ cells were transfected by electroporation with 3 μg vector. Clones of cells were generated by dilution. For mutation screens, genomic DNA of single-cell clones was isolated and the CRISPR/Cas9 target region was amplified by PCR. Products were resolved by polyacrylamide gel electrophoresis. Genomic DNA of clones of cells with putative indel mutations was PCR amplified and cloned into pGL3 vector (Promega) and subjected to Sanger sequencing. The top five potential off-targets in DNA regions were tested for the off-target effects by PCR amplification and polyacrylamide gel electrophoresis analysis. Expression of all genes with potential off-target sites in the coding regions was tested by qPCR in the clones. No off-target effects were detected. From 37 clones of cells, we identified 7 clones of cells with indel mutations at the HRE and chose 10 non-altered clones as controls.

### Allele-specific assays

To identify samples heterozygous for the common and rare alleles at rs35252396 and rs11604, DNA from cell lines and primary tubular cells was genotyped using customized Taqman assays (Life Technologies). All primers, probes and conditions used are available on request. For allele-specific assays DNA from FAIRE and ChIP experiments as well as cDNA from primary renal tubular cell cultures was used. Genomic DNA from untreated samples from the same experiments was used in serial dilutions as an input control. Homozygous cell lines for both alleles were used as positive controls (rs35252396: AC/AC-Caki1 cells, CG/CG-786-O cells; rs11604: T/T-RCC L15, C/C-RCC L13) in all allele-specific assays. Data were analysed using the TaqMan Genotyper Software V1.3 (Life Technologies). For both assays, the mean ratio of minor allele/major allele (FAM/VIC) for the input DNA was arbitrarily set to 1 and the ratios of DNA from assays (FAIRE; ChIP or cDNA) were normalized to input DNA ratios. For the intragenic SNP, rs11604 allelic ratios of cDNA from DMOG-treated primary tubular cells were compared with the allelic ratio of the corresponding untreated control cells.

### Tumour samples

A total of 453 renal tumours and corresponding normal renal tissue were collected from the archives of the Department of Pathology, University of Erlangen-Nürnberg, for tissue microarray construction. The tumour collection consists in part of older samples (before 2008) that were collected anonymously. The local Ethical Committee has approved the use of these samples for this study without informed consent. For more recent samples, informed consent was provided by the patients. The entire study has been approved by the local Ethical Committee at the University of Erlangen-Nürnberg and specimens were collected in accordance with the World Medical Association Declaration of Helsinki. Details on tissue microarray (TMA) composition and tumour characteristics have been published previously^55^. In short, archival FFPE tissues were reclassified according to the 2004 World Health Organization classification of renal tumours and the 2002 tumour node metastasis (TNM) classification by two uropathologists. One representative punch from each tumour and from corresponding normal tissue was transferred to a new block for TMA construction.

### Immunohistochemistry

Immunostainings were conducted on paraffin-embedded tissue arrays as described earlier^3^. Antibodies were anti-MYC (1:200, rabbit monoclonal (Y69), Abcam, ab32072), anti-HIF-1α (1:10,000, rabbit polyclonal, Cayman Chemicals, Cay10006421) and anti-HIF-2α (1:10,000, rabbit polyclonal, PM8), and were applied after an antigen retrieval procedure (Dako). Stainings were analysed by two researchers blinded for tumour phenotype and results of other stainings. Stainings were scored according to intensity of nuclear staining (0–4) and per cent of positive cells (0–100%) using an immunoreactive score (IRS) according to Remmele and Stegner^56^. HIF and MYC stainings were then divided into four categories: no (IRS=0), low (IRS 1–3), medium (IRS 4–8) or high (9–12) levels of immunoreactivity. A small proportion of samples from the TMA (<5%) could not be analysed due to the lack of tissue or due to the presence of normal kidney tissue at the respective position on the TMA.

### TCGA data

RNAseqV2 level 3 data sets with normalized expression values from clear cell, papillary and chromophobe renal cell carcinoma tumours were downloaded from the TCGA website (http://cancergenome.nih.gov/)^39^,^40^,^41^. Samples with matching normal and tumour data were identified and a similar number of paired data sets was processed for each tumour subtype (KIRC: batch 82; *n*=22; KIRP: batches 51, 71, 162, 281, 299, 386; *n*=31; KICH: batch 226; *n*=25).

### Mouse expression data sets

Raw data from expression arrays from kidney of for Pax8-rtTA;LC-1;Vhl-floxed mice and control littermates were generated by N.M. Farsijani and V. Haase and downloaded from http://www.ncbi.nlm.nih.gov/geo (GSE54172)^57^. After quantile normalization, signals for Myc, Pvt1 and Egln3 were compared between tubular-specific *VHL* knockout and control mice.

### Data availability

The Chip-seq data that support the findings of this study have been deposited in the gene expression omnibus under accession codes GSE28352, GSE67237 and GSE78113. The Capture-C data generated for this study are available under accession code GSE84444.DNA accessibility data referenced in this study are available from the ENCODE project in the gene expression omnibus under accession codes GSE35239 and GSE32970. Mouse expression data are available in the gene expression omnibus under accession code GSE54172. TCGA data from renal tumours were downloaded from the TCGA website (http://cancergenome.nih.gov/). All remaining data are contained within the Article and Supplementary Information files or available from the author on request. Data analysis—statistical analyses for RNA expression were performed using a one-sample *t*-test, a Student's *t*-test or a Mann–Whitney *U*-test if applicable using GraphPadPrism Version 5.00 (GraphPad Software Inc). IRSs and eQTL analyses were evaluated using the *χ*^2^-test (IBM SPSS Statistics 21).

## Additional information

**How to cite this article**: Grampp, S. *et al*. Genetic variation at the 8q24.21 renal cancer susceptibility locus affects HIF binding to a MYC enhancer. *Nat. Commun.* **7**, 13183 doi: 10.1038/ncomms13183 (2016).

## Supplementary Material

Supplementary Information Supplementary Figures 1-16, Supplementary Tables 1-5
