# Supplementary material for: Genetic variation at the 8q24.21 renal cancer susceptibility locus affects HIF binding to a MYC enhancer
Source: Nat Commun. 2016 Oct 24;7:13183. doi: 10.1038/ncomms13183 (PMC5079059; doi:10.1038/ncomms13183)
Supplement: Supplementary Information — Supplementary Figures 1-16, Supplementary Tables 1-5 [file ncomms13183-s1.pdf]

Supplementary Figure 1 **MYC protein in RCC subtypes**

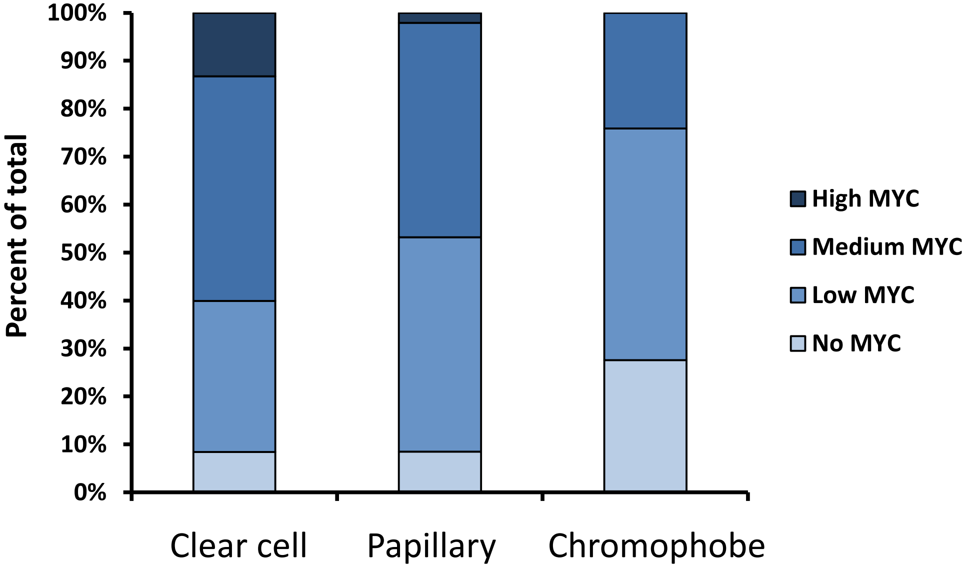

Supplementary Figure 1: Renal tumors from a tissue microarray (n=453) were stratified according to the immunoreactive score obtained for MYC staining; no (0), low (1-3), medium (4-8) or strong (9-12) staining. Fractions of MYC signals are shown for each renal cancer subtype. X^2-test; $p<0.001$ for MYC staining in ccRCC.

Supplementary Figure 2 MYC and PVT1 regulation by DMOG in different cell lines

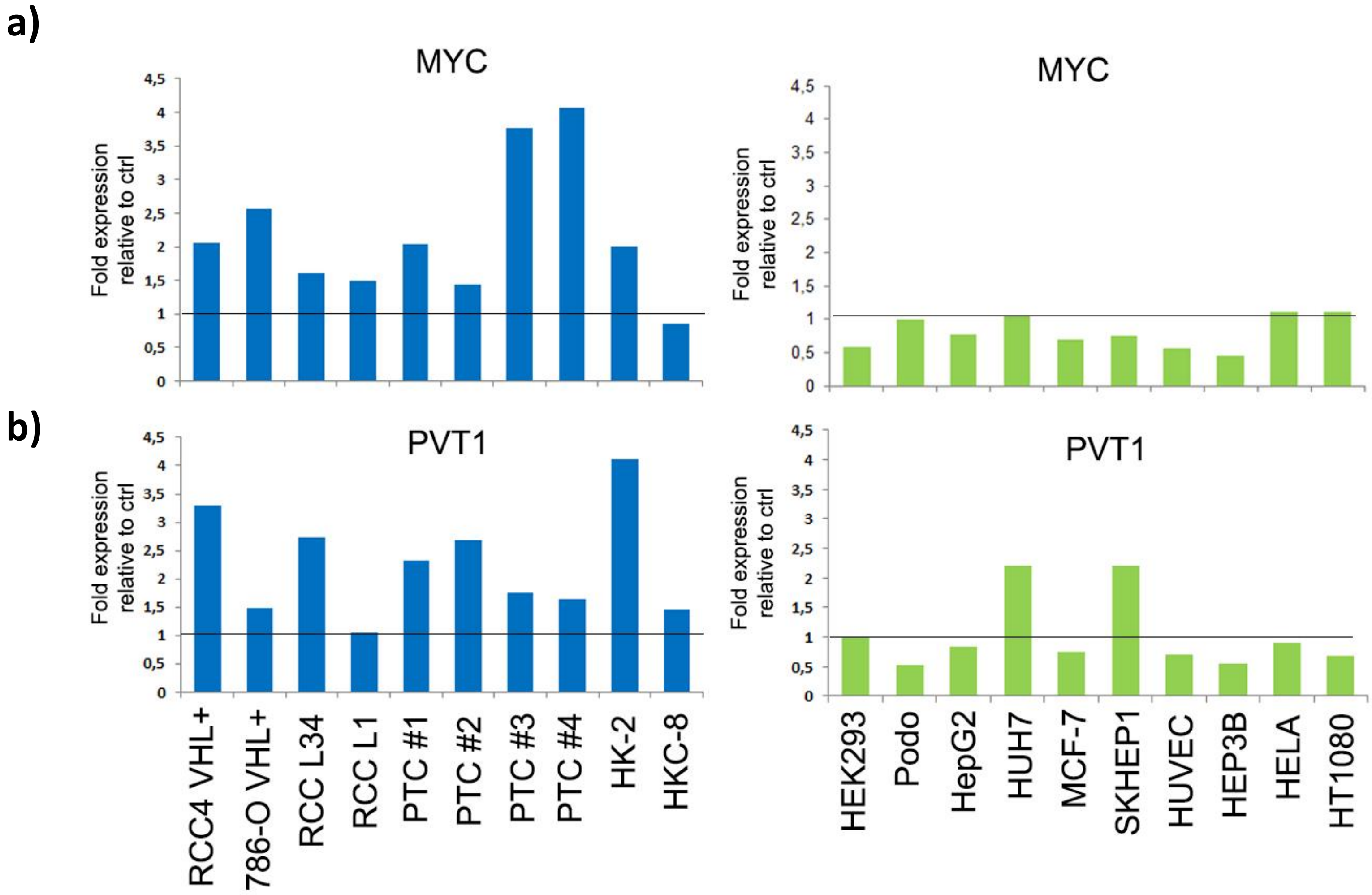

Supplementary Figure 2: Relative expression levels (compared to vehicle control, ctrl) of **a)** MYC and **b)** PVT1 RNA in a collection of ten VHL-competent renal tubule derived cell types (RCC: RCC4, 786-O, RCC L34, RCC L1, primary renal tubular (PTC) and immortalized renal tubular (HK-2, HKC-8) cells, blue) and ten non-renal tubule derived cell types (HEK293, podocytes and non-renal cells, green) exposed to 1mM DMOG for 16h.

Supplementary Figure 3 MYC expression in mouse kidneys with conditional deletion of VHL in tubular cells

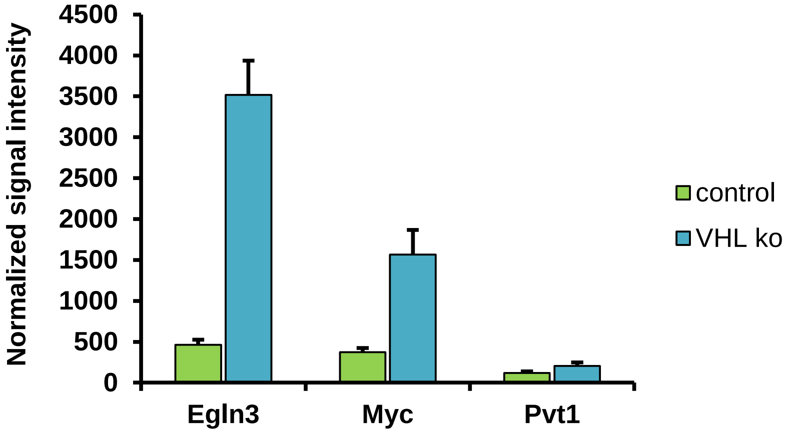

Supplementary Figure 3: Expression levels of Egln3, Myc and Pvt1 RNA in mouse kidneys with or without PAX8-Cre mediated deletion of VHL in renal tubular cells. Bars show mean ±SD. Data was retrieved from GEO data set: GSE54172 (please see Method section for details).

Supplementary Figure 4 HIF knock down in RCC cells: MYC and PVT1 expression

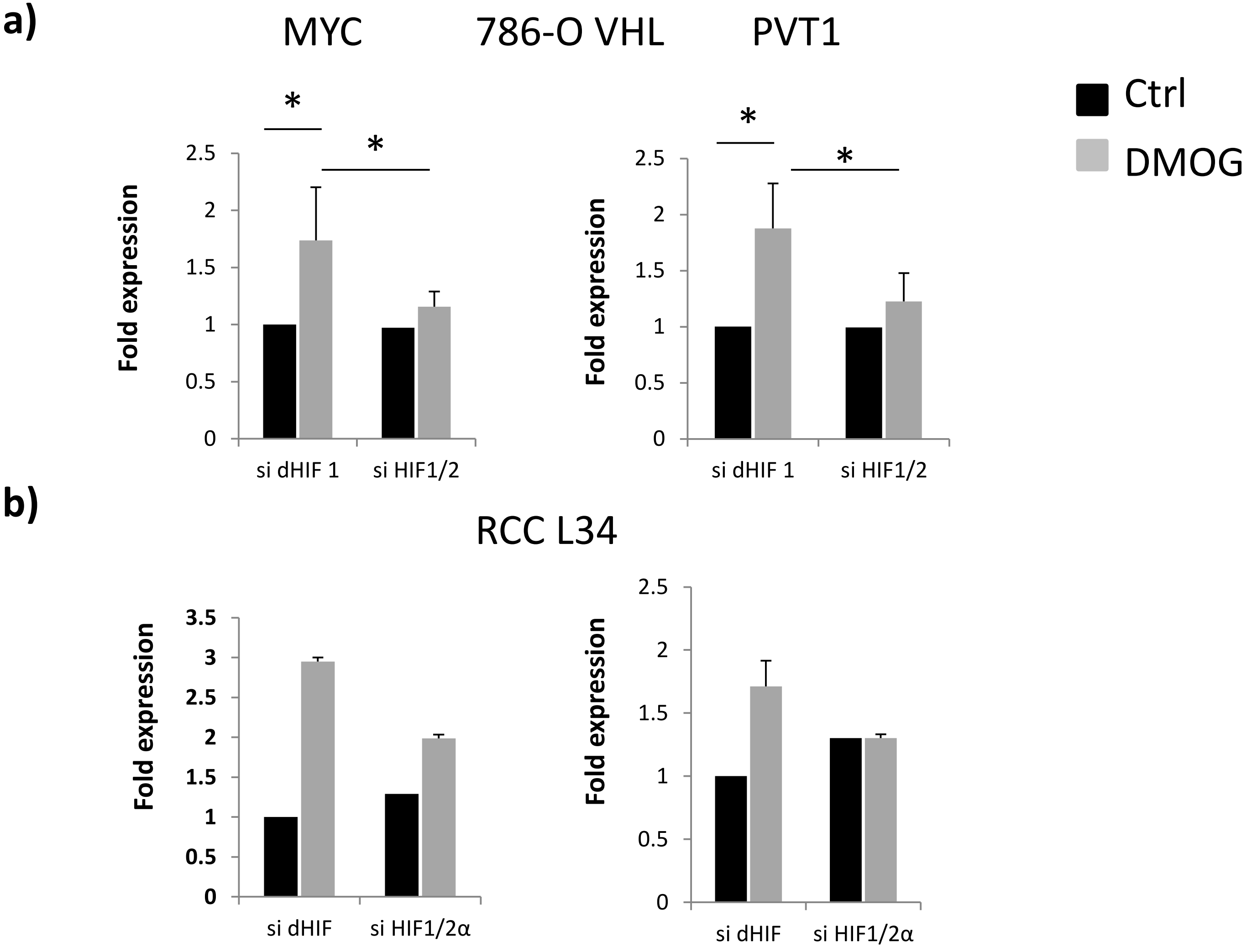

Supplementary Figure 4: Expression levels of MYC and PVT1 RNA in HIF-α depleted (si HIF1/2a, siRNA against HIF1/2a) or control transfected (si dHIF, siRNA against drosophila HIF) **a)** 786-O and **b)** RCC L34 VHL-competent cells exposed to vehicle (black) or 1mM DMOG (grey) for 16h. Data is from four independent knock down experiments (786-O) or from one experiment (RCC L34) performed in triplicate qPCRs. Bars show mean ±SD. *, $p<0.05$, one-sample t-test compared to the indicated samples.

Supplementary Figure 5 FAIRE at 8q24.21 HIF-binding site

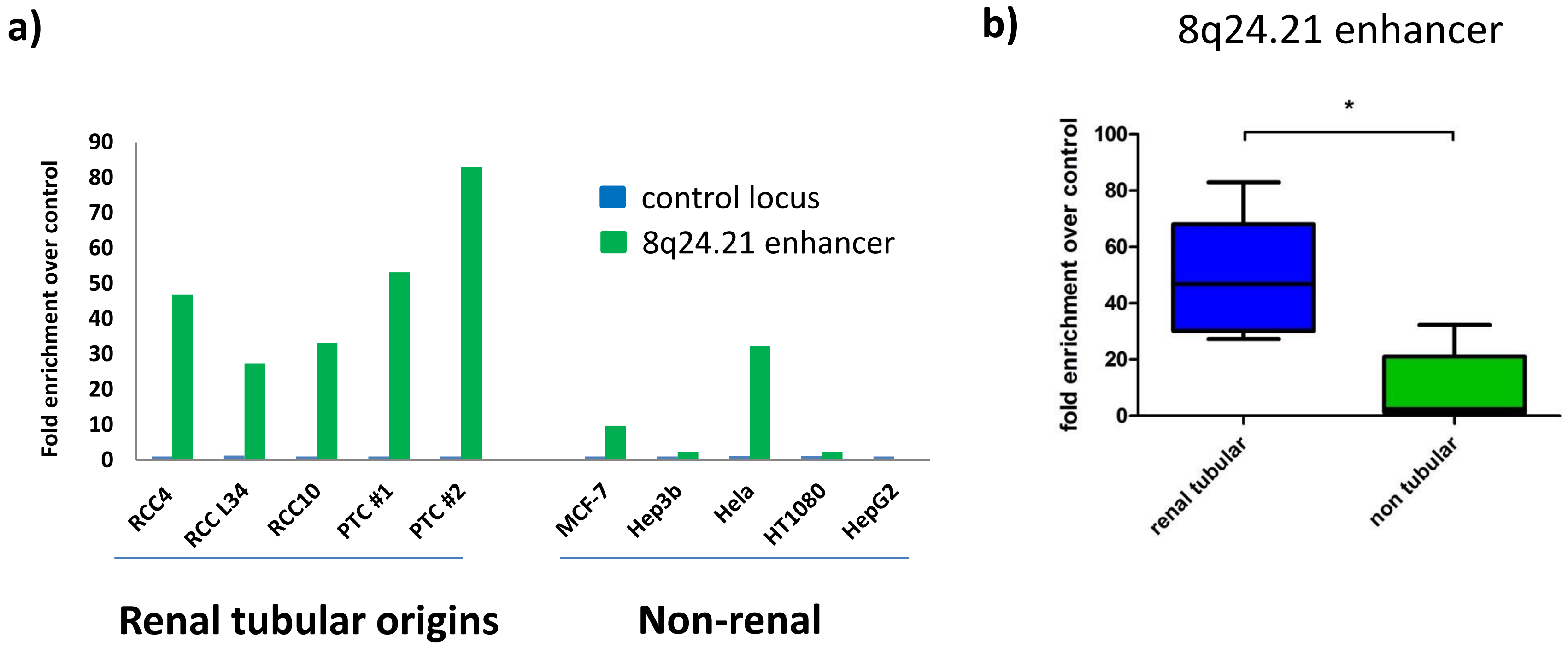

Supplementary Figure 5: a) FAIRE qPCR in different cell lines shows chromatin accessibility at the 8q24.21 HIF-binding site (green) predominantly in cells from tubular origins. A locus on chromosome 8 with no open chromatin conformation (blue) was used as negative control. Data shows mean from one experiment each performed using triplicate qPCRs. **b)** Box-whisker blots of data in a). Horizontal lines represent median and whiskers show the 5 and 95% percentiles; *, Mann-Whitney U test, $p < 0.05$.

Supplementary Figure 6 Capture-C assays at 8q24.21 HIF-binding site

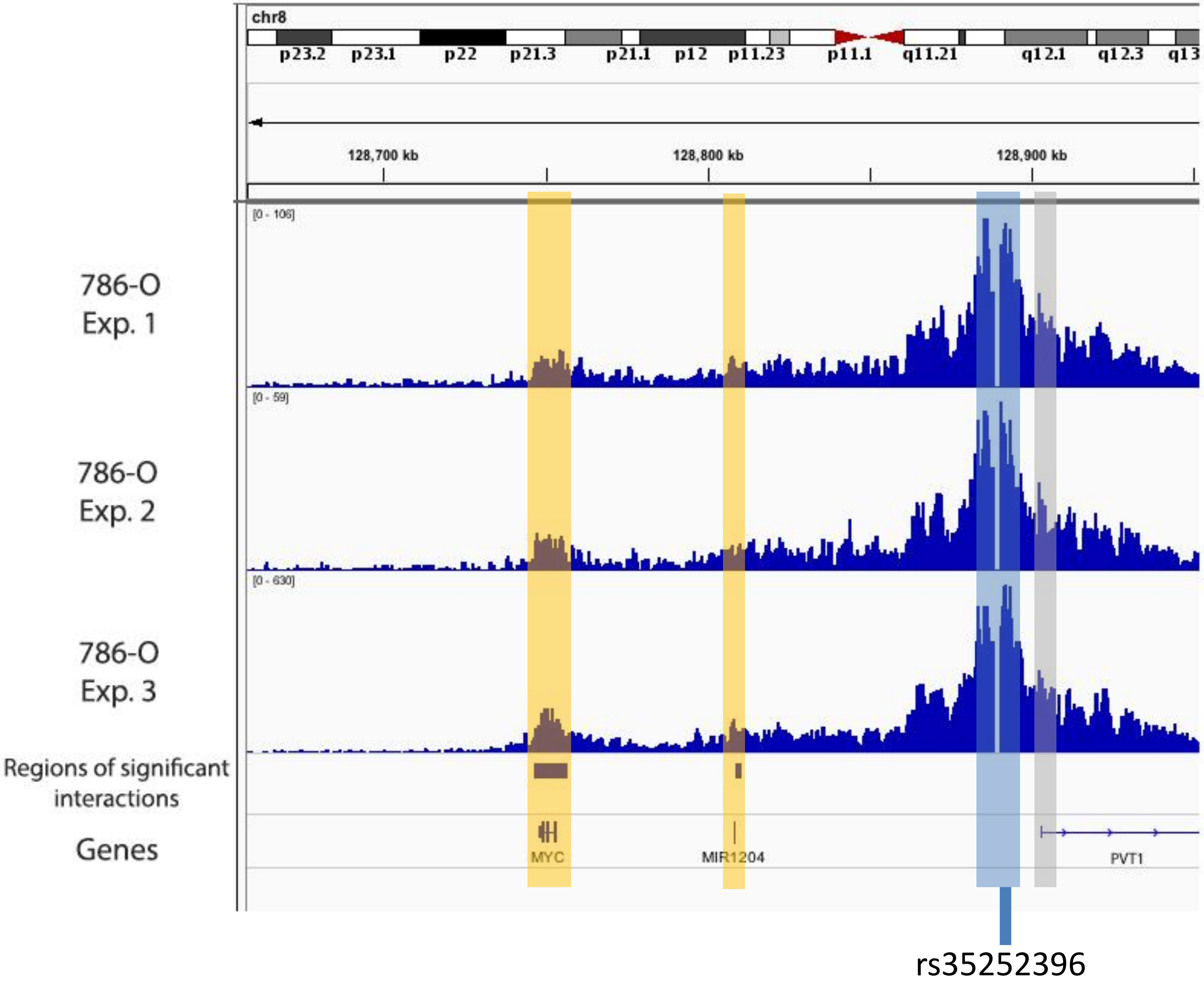

Supplementary Figure 6: Capture-C data from three independent experiments (Exp. 1 –Exp. 3) performed in 786-O renal cancer cells. Number of reads are indicated at the left side of each track. Bars below the tracks indicate regions of significant interaction with the rs35252396 associated enhancer (viewpoint, highlighted in blue) as determined in the combined analysis. There are significant interactions with the *MYC* and the *MIR1204* loci (highlighted in orange), however Interactions with the *PVT1* promoter (highlighted in grey) did not reach statistical significance using a background model that takes into account the distance dependent decay effect of interactions (regions close to the region of interest are more likely to interact than more distant regions).

Supplementary Figure 7 Genome editing at the 8q24.21 enhancer in 786-O RCC cells

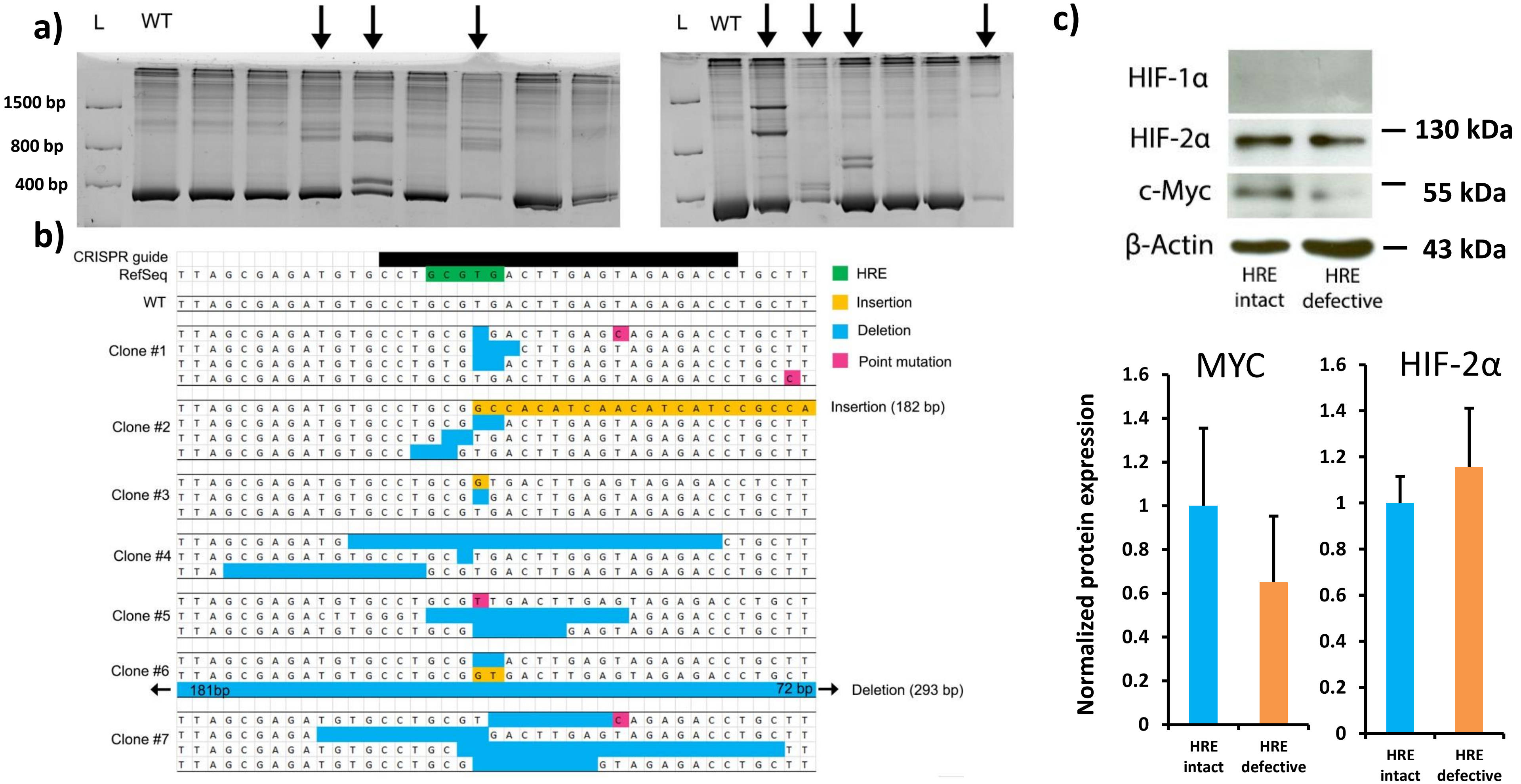

Supplementary Figure 7: a) Representative polyacrylamide gel (PAGE) analyses of PCR products (329bp amplified from the 8q24.21 enhancer locus) from single clones of cells. Single clones of cells were derived from a pool of 786-O cells transfected with CRISPR/Cas9 and a guide RNA targeting the HRE (green in b). Arrows mark products with an altered genetic sequence. L: ladder, WT: wild type parental cells. **b)** Sanger sequencing of PCR products from a) ligated into the pGL3 promoter vector. Seven PAGE positive single clones of cells exhibit indel mutations centered on the HRE. Position of the guide RNA targeting the HRE is indicated as a black bar in the top row. WT: wild type 786-O cells. **c)** Protein levels of MYC are reduced in HRE defective clones of cells. Representative western blot from a HRE-intact and a HRE-defective clone (top). Densitometric signals for MYC and HIF-2α were normalized to the respective actin B signal and to the average signal of HRE intact cells. Bars show mean ±SD. n=4 for HRE intact and HRE defective cells.

Supplementary Figure 8 Control ChIP experiments in 786-O HRE intact or defective cells

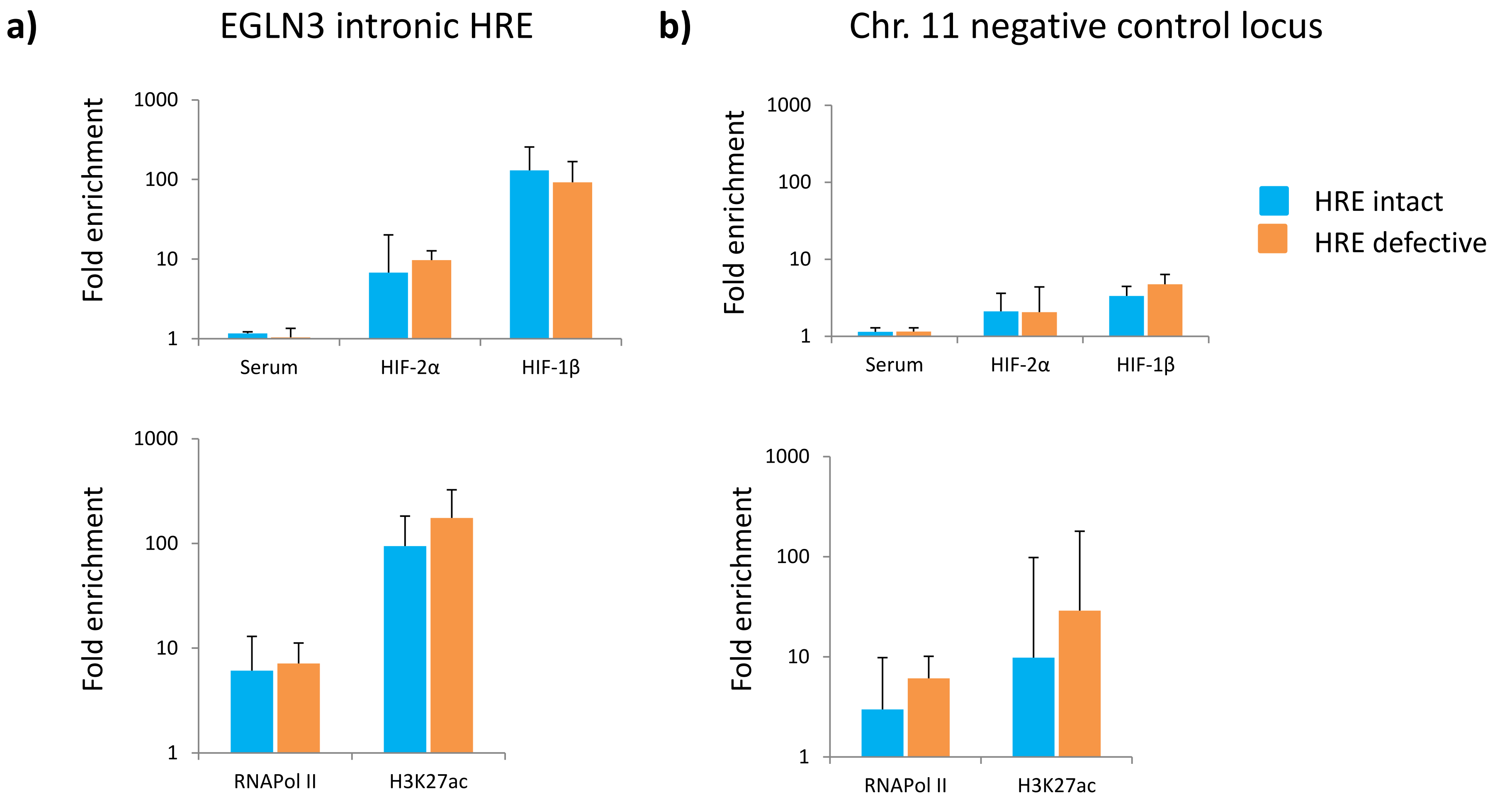

Supplementary Figure 8: ChIP qPCR against **a)** the HIF-binding *EGLN3* intronic HRE on chromosome 14 and **b)** a negative control site on chromosome 11. Values are from 3 different clones of cells per group and normalised to values obtained for serum control immunoprecipitations. Bars show mean ±SD on a logarithmic scale.

Supplemental Figure 9 8q24.21 enhancer reporter assays

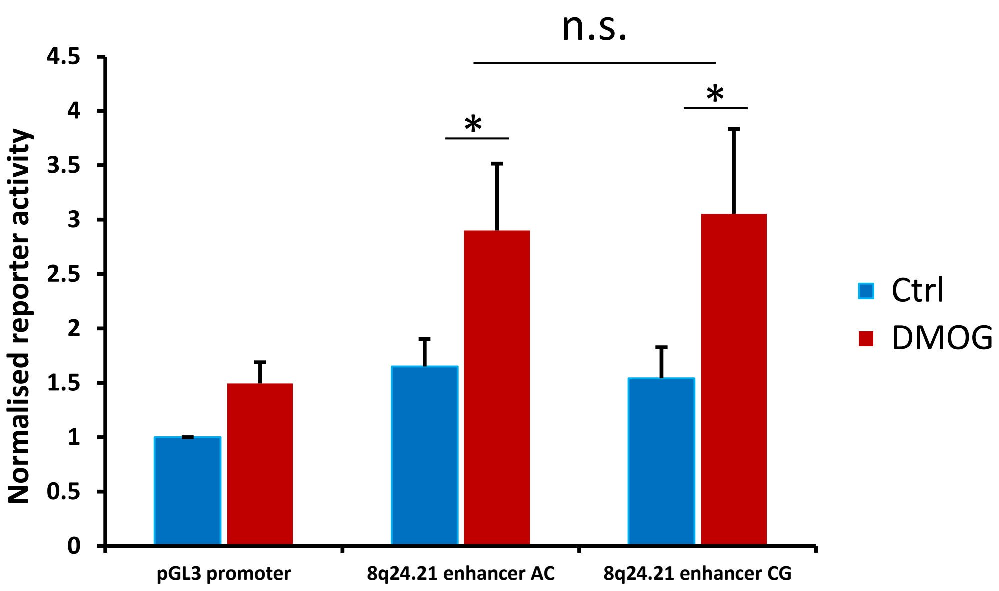

Supplemental Figure 9: Reporter assay using a 329bp sequence (hg19: chr8:128889122-128889450) containing the 8q24.21 HIF-binding site and rs35252396 performed in Hela cells. The two haplotypes (AC and CG) were PCR amplified from genomic DNA isolated from homozygous cell lines and inserted into the pGL3 promoter vector. Reporter plasmids were transfected into Hela cells using Xtreme Gene transfection reagent (Roche) and cells were stimulated with vehicle (control, ctrl) or 1mM DMOG for 16h. Raw data from luciferase measurements were normalized to activity of cotransfected β-galactosidase and to values for pGL3 promoter control. Results are from six independent experiments. Bars show mean ±SD. *, Student‘s t test, $p < 0.05$ when comparing results from DMOG treated cells versus respective control untreated cells. n.s., non significant.

Supplementary Figure 10   Allele-specific HIF-binding in RCC L13 cells

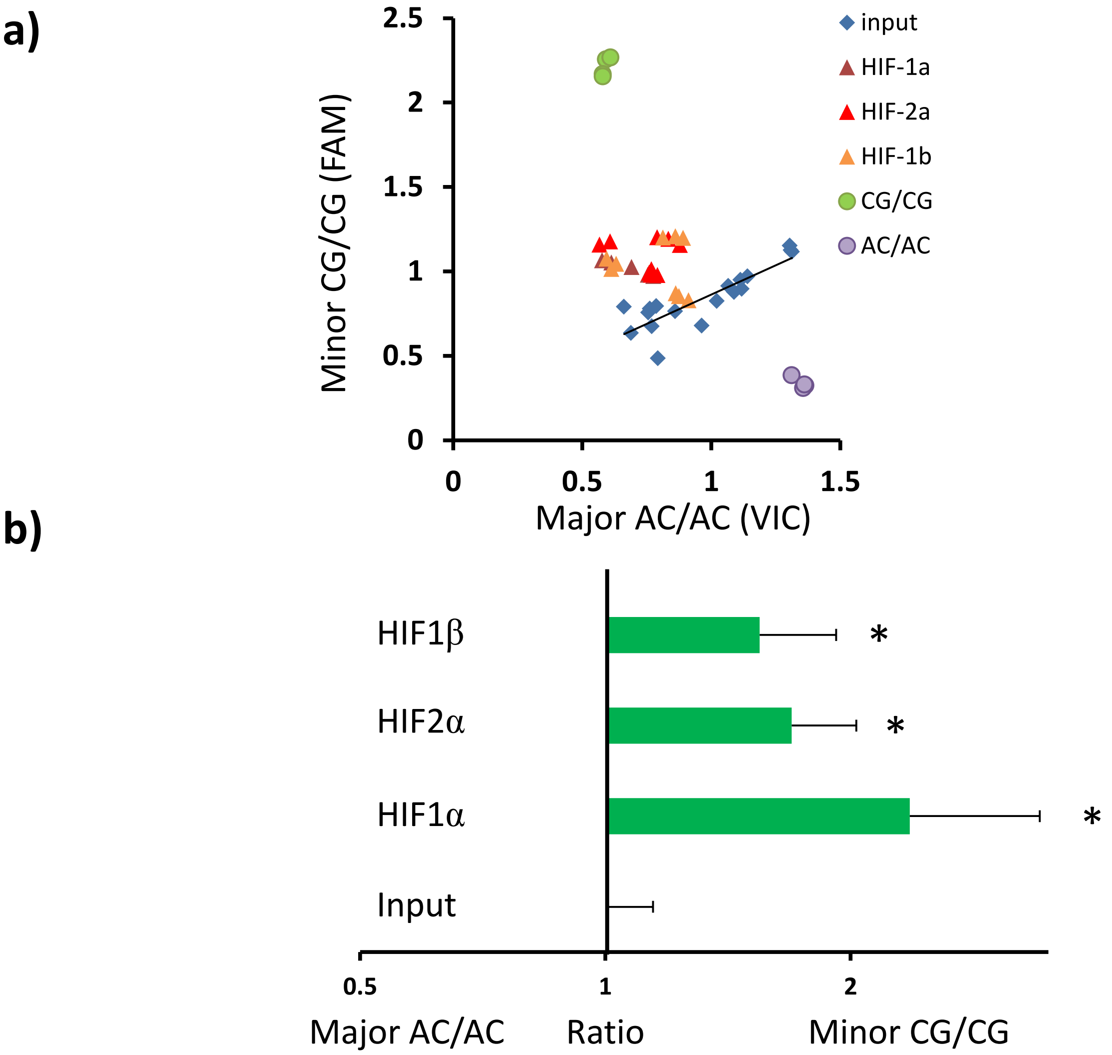

Supplementary Figure 10: a) Genotype specific assay for rs35252396 using DNA captured by ChIP using antibodies against HIF1α, HIF2α and HIF1β in RCC L13,cells. Input DNA was used as a control and DNA from homozygous cell lines was used for positive controls for the homozygous alleles (AC/AC and CG/CG). A shift of the qPCR signals towards the minor allele CG indicates enrichment of the risk allele. Data show fluorescence signals for FAM (AC) and VIC (CG) and are from three independent ChIP experiments. **b)** Ratios of qPCRs signals from a). The mean ratio of minor allele / major allele for the input was arbitrarily set to 1. Data is mean ±SD. *, one-sample t-test, $p<0.05$.

Supplementary Figure 11 Allele-specific active chromatin at the 8q24.21 RCC enhancer

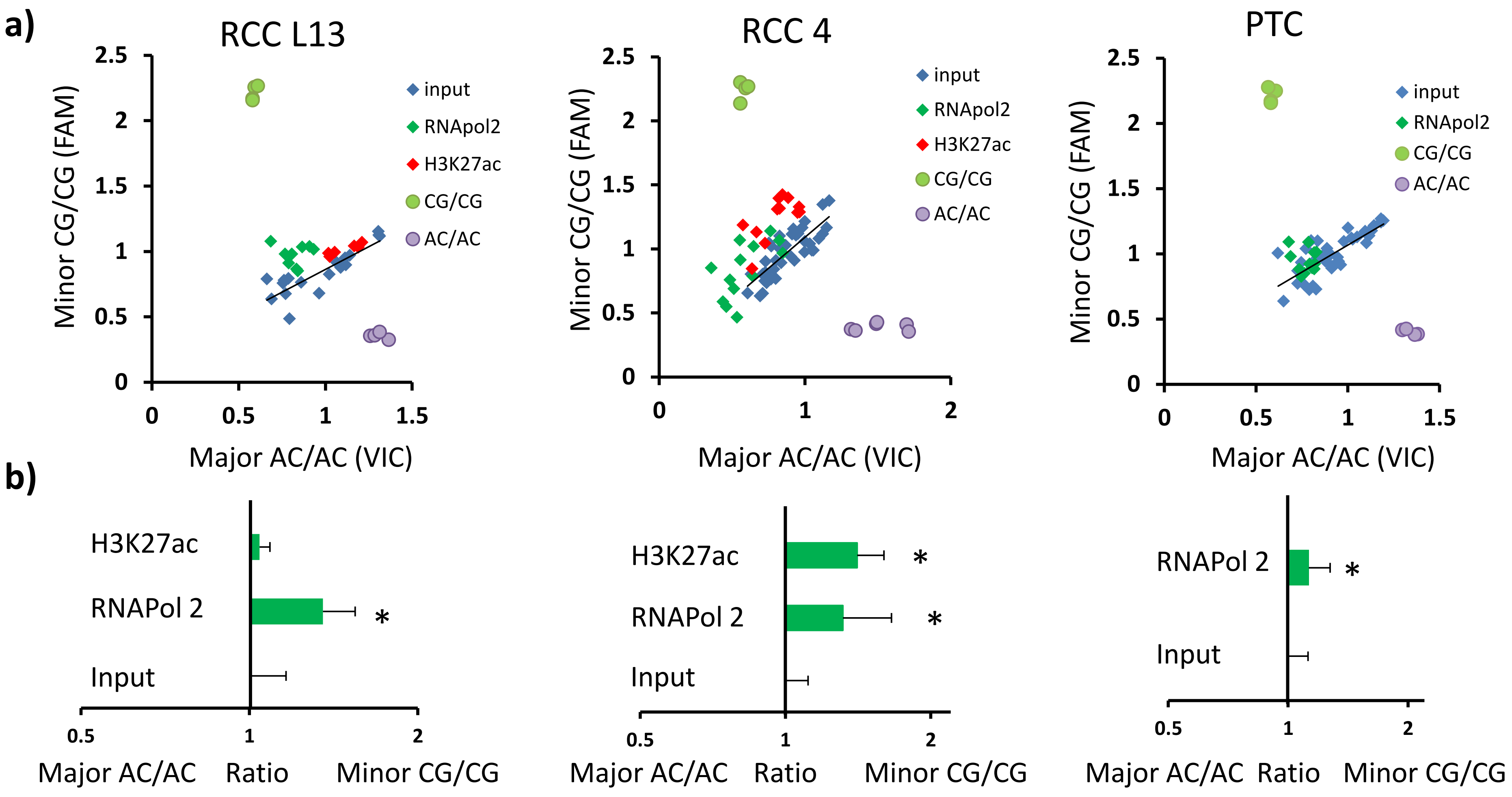

Supplementary Figure 11: a) Genotype specific assay for rs35252396 using DNA captured by ChIP using RNApol 2 and H3K27ac antibodies in RCCL13, RCC4 and PTC cells. A shift of the qPCR signals towards the minor allele CG indicates enrichment of the risk allele in active chromatin. Data are from three independent ChIP experiments (RCC L13 and RCC4). Homozygous controls for RCC L13 are the same as in Fig. S8a because these samples were analyzed in the same qPCR experiment. For PTC, only the RNApol2 antibodies were used since these were samples from historical ChIP experiments in which H3K27ac antibodies were not used. Data is from a) three experiments using PTC from three individuals. **b)** Ratios of qPCRs signals from a). The mean ratio of minor allele / major allele for the input was arbitrarily set to 1. Data is mean ±SD. *, one-sample t-test, $p<0.05$.

Supplementary Figure 12 **Sequencing PCR amplicons at the 8q24.21 enhancer from RCC4 ChIP experiments**

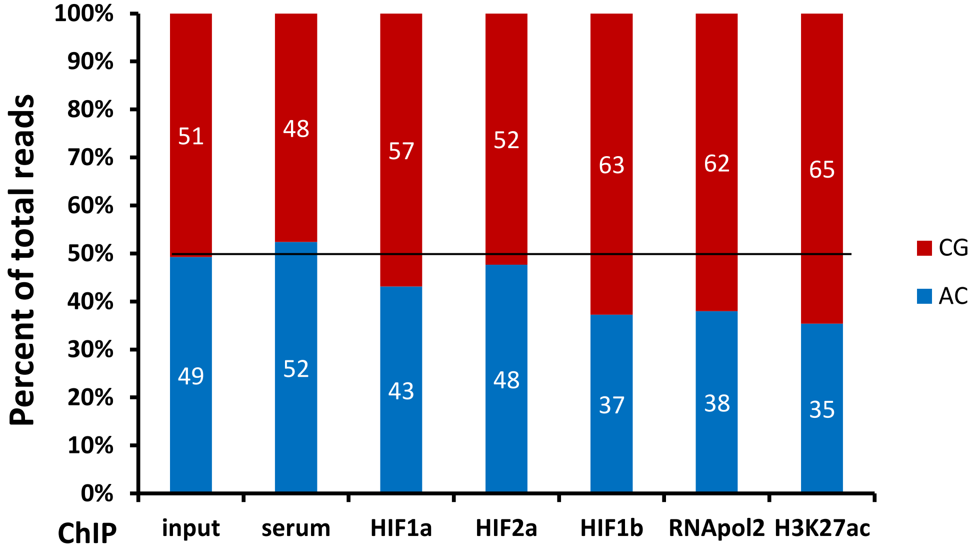

Supplementary Figure 12: Allelic imbalance in ChIP samples from RCC4 cells at the 8q24.21 enhancer. A 329bp sequence (hg19: chr8:128889122-128889450) was PCR amplified using barcoded PCR primers. PCR products were subjected to amplicon sequencing (MiSeq sequencing system, Illumina) and amplicons for each immunoprecipitation were analysed for the presence of the protective AC or the risk CG allele. The relative abundance of each allele in the ChIP samples is shown. Data were generated from one ChIP experiment in RCC4 cells.

Supplementary Figure 13   Allele-specific open chromatin at the 8q24.21 RCC enhancer in RCC L13

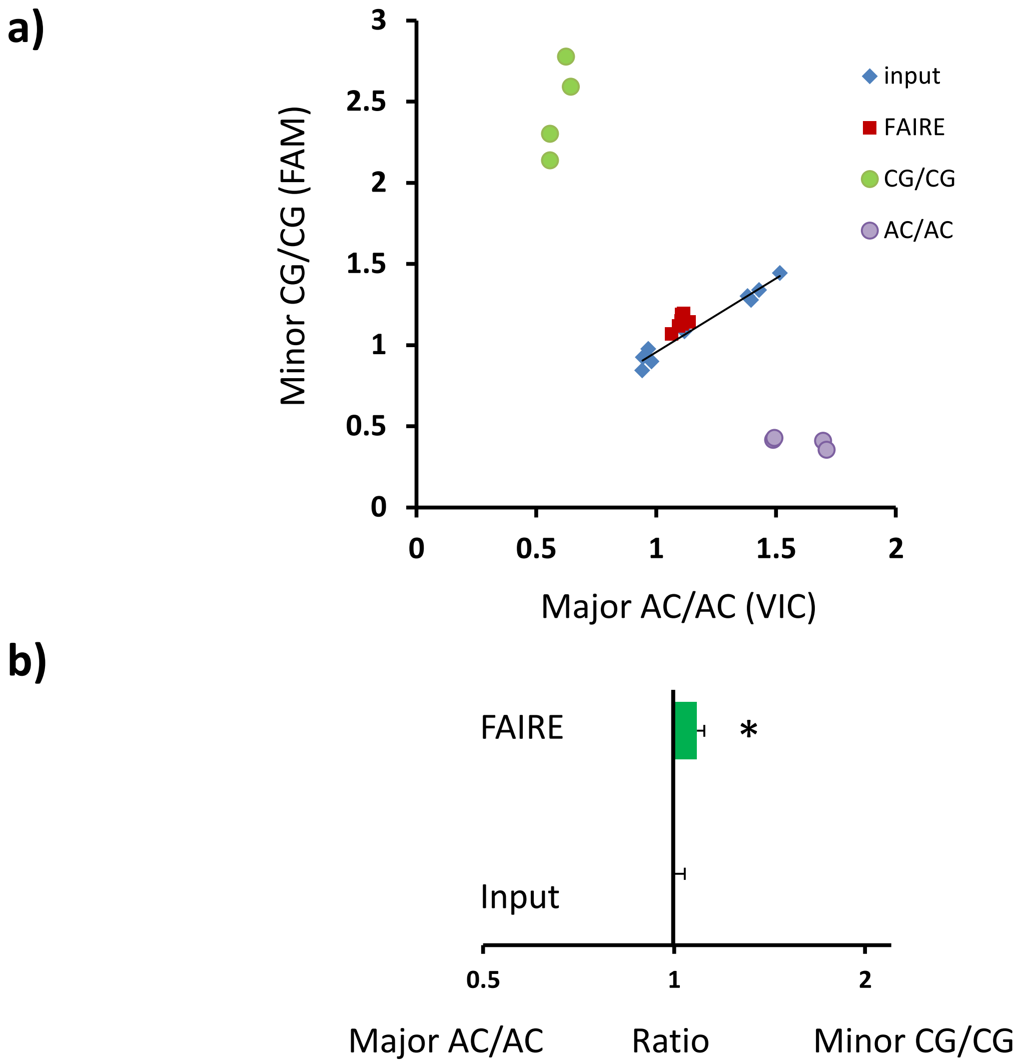

Supplementary Figure 13: a) Allele specific qPCR for rs35252396 using FAIRE and input DNA samples from RCCL13 cells. **b)** Ratios of qPCRs signals from a). The mean ratio of minor allele / major allele for the input was arbitrarily set to 1. Data is mean ±SD. *, one-sample t-test, $p<0.05$.

Supplementary Figure 14 SNP rs11604 in PVT1 coding region

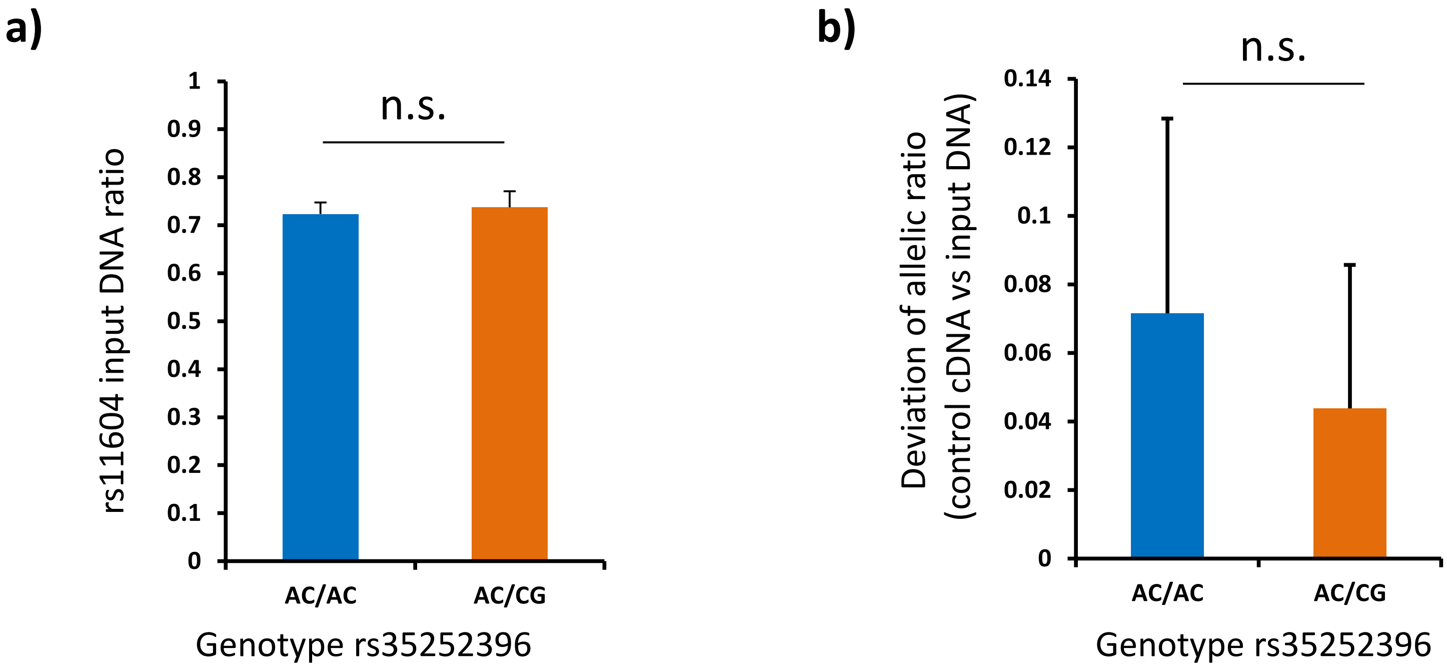

Supplementary Figure 14: Allele specific qPCR for rs11604 using **a)** input DNA or **b)** control cDNA samples from primary tubular cells . Cells were stratified according to the gentopye at rs35252396 (AC/AC n=4, AC/CG n=8). We measured the allelic ratio for rs11604 of qPCR signals (FAM/VIC) from input DNA and cDNA derived from control cells. Figure b) shows the mean deviation of the cDNA ratio from the input DNA ratio for rs11604. There is no significant (n.s., non significant) difference between input DNA or the mean deviation from the input ratio in control cDNA ratios between the two groups . Data is mean ±SD.

Supplementary Figure 15 **Genotype-expression correlation TCGA KIRC data**

a)

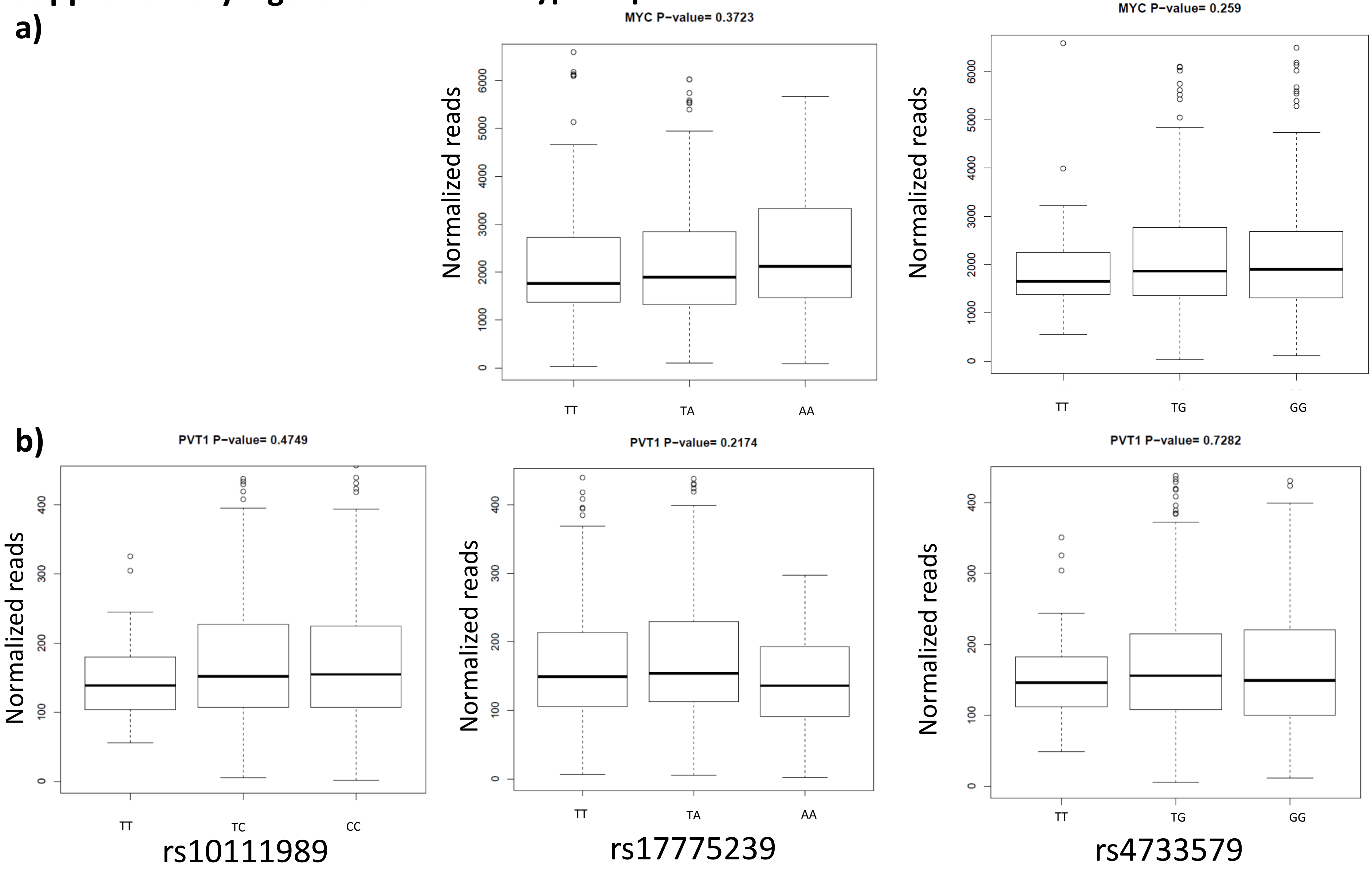

Supplementary Figure 15: Genotype-expression correlation of SNPs in the 8q24.21 region that are in LD with rs35252396. **a)** Correlation with MYC RNA expression and **b)** correlation with PVT1 RNA expression in the TCGA KIRC data. Chi square test was used to calculate p-values which are indicated at the top of each graph. LD with rs35252396: rs10111989: r^2=0.33, D'=0.98; rs17775239: r^2=0.41, D'=1; rs4733579: r^2=0.46, D'=0.98. Risk alleles for the SNPs were confirmed from data of a meta-analysis of UK and NCI cohorts (reference 49) (C for rs10111989, $p<0.05$; A for rs17775239; G for rs4733579, $p<0.01$)

Supplementary Figure 16 **Western blots of MYC, HIF-2α and β-actin in 786-O cells**

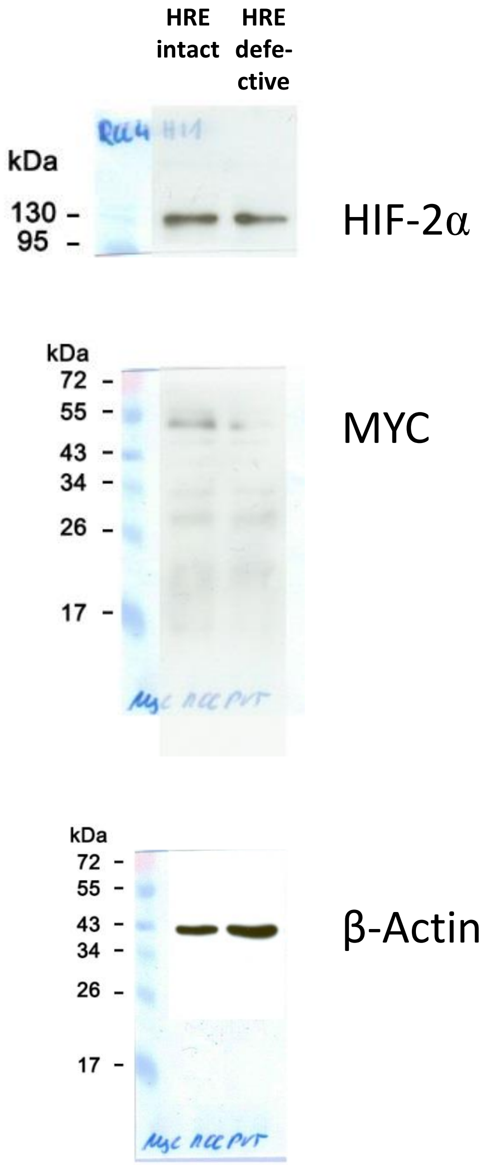

Supplementary Figure 16: Uncropped scans of representative Western blots (from Supplementary Figure 7) for HIF-2α, MYC and β-Actin using lysates of 786-O clones of cells with an intact or a defective HRE, respectively.

Supplementary Table 1

**ChIP Primers**	**fw 5´-3´**	**rev 5´-3´**
PVT1 HIF Bind	TGCTGTCATCGCTTTCATTT	TAGCTTGCCCAAGGATTCTG
EGLN3	AGTGTCCGTTCCCAGCTCAG	TAGGCACAGTAAACAGGCC
Control	GGATCACCAGGTGTATTCGG	CATCCAAGGGGGATACACCA

Supplementary Table 2

**siRNA**	**sense**	**antisense**
dHIF	CCUACAUCCCGAUCGAUGAtt	UCAUCGAUCGGGAUGUAGGtt
HIF-1α	CUGAUGACCAGCAACUUGAtt	UCAAGUUGCUGGUCAUCAGtt
HIF-2α	CAGCAUCUUUGAUAGCAGUtt	ACUGCUAUCAAAGAUGCUGtt

Supplementary Table 3

**Expression Primers**	**fw 5´-3´**	**rev 5´-3´**
PVT1	TGTAAGACCCCGACTCTTCC	GCTGTATGTGCCAAGGTCAC
MYC	CTCTCCGTCCTCGGATTCT	CAACATCGATTTCTTCCTCATC
HPRT	GACCAGTCAACAGGGGACAT	AACACTTCGTGGGGTCCTTTTC
EGLN3	GGCCATCAGCTTCCTCCTG	GGTGATGCAGCGACCATCA

Supplementary Table 4

**FAIRE Primers**	**fw 5´-3´**	**rev 5´-3´**
PVT1	TGTAAGACCCCGACTCTTCC	GCTGTATGTGCCAAGGTCAC
Control	AGCAAAGCAGAATGGAAGGA	CCCCACTGTGGAGTTTGTCT

Supplementary Table 5

**Cloning Primers**	**fw 5´-3´**	**rev 5´-3´**
PVT1 enhancer	CATGGTACCGGATGGGGACTAAGTGGGAT	CATGGTACCAAGCTAGATGGCTGCCAAAG
